# Mechanisms of Direct Electron Transfer Governed by Redox‐Active Conductive Carrier with Superior Wettability in Anaerobic Biofilms

**DOI:** 10.1002/advs.202516258

**Published:** 2025-11-29

**Authors:** Junli Tian, Xiaoyuan Zhang, Lulu Xing, Bin Ji, Jinfeng Lu, Yu Liu

**Affiliations:** ^1^ Engineering Laboratory of Low‐Carbon Unconventional Water Resources Utilization and Water Quality Assurance College of Environmental Science and Engineering Nankai University Tianjin 300350 China; ^2^ Key Laboratory of Pollution Processes and Environmental Criteria Ministry of Education Nankai University Tianjin 300350 China; ^3^ Department of Water and Wastewater Engineering School of Urban Construction Wuhan University of Science and Technology Wuhan 430065 China

**Keywords:** anaerobic biofilm, cytochrome c, direct electron transfer, redox‐active conductive carrier, redox‐active cycle, surface wettability

## Abstract

Biofilms cultivated on conductive carriers emerge as promising systems for enhancing anaerobic wastewater treatment, while the underlying electron transfer mechanisms remain insufficiently elucidated. Herein, a redox‐active conductive carrier composed of carbon felt functionalized with tannic acid‐modified iron‐biochar (TA‐FeBC) with superior wettability is engineered to regulate direct electron transfer (DET) at the anaerobic biofilm‐carrier interface. It possesses a redox potential significantly lower than CO_2_/CH_4_ and an exceptional electron‐donating capacity of 16.6 µmol e^−1^ g^−1^, collectively creating a strong thermodynamic driving force for DET‐driven methanogenesis. In contrast to conventional direct interspecies electron transfer (DIET), the redox‐active conductive TA‐FeBC carrier may act as an exogenous electron donor, channeling electrons directly into anaerobic biofilm through cytochrome c (CytC)‐mediated pathway. Notably, the novel mechanism reduces the electron transfer resistance of anaerobic biofilm by over 50‐fold compared to anaerobic suspended sludge. The higher flat‐band potentials of anaerobic biofilm (−0.131 V) compared with the redox‐active conductive TA‐FeBC carrier (−0.301 V) favors a steep redox gradient, enabling *Methanobacterium* to directly harvest electrons from the carrier, as evidenced by a stable 110 µA cm^−2^ cathodic current. This study provides the first integrated experimental evidence for CytC‐mediated DET governed by an engineered conductive carrier, offering new avenues for rational design of redox‐active carriers in bioelectrochemical and anaerobic systems.

## Graphical abstract

A redox‐active conductive carrier of tannic acid‐modified iron‐biochar with superior wettability is demonstrated to regulate direct electron transfer at the anaerobic biofilm‐carrier interface by enhancing the cytochrome c‐mediated pathway. For the first time, integrated evidence from spectroscopy, electrochemical analyses, and metatranscriptomics provides mechanistic insight into redox‐active conductive carrier assisted methanogenesis.

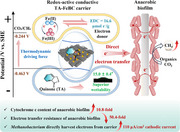

## 1 Introduction

Anaerobic wastewater treatment has been widely recognized as a sustainable technology for energy recovery from wastewater,^[^ ^1^ ^]^ while its practical application was hindered by limited treatment efficiency and operational stability.^[^ ^2^ ^]^ Compared to suspended sludge, biofilm formed on solid carriers could offer a favorable microenvironment for microbial colonization and activity, providing superior settleability, enhanced cell‐to‐cell contact, and reduced electron transfer distances.^[^ ^3^ ^]^ The redox activity and electrical conductivity of the biofilm‐carrier interface are critical in determining its electron‐accepting and donating capabilities during extracellular electron transfer.^[^ ^3^, ^4^ ^]^ Therefore, enhancing electron transfer is a key strategy for enhancing anaerobic biofilm performance.

To this end, conductive materials have gained increasing attention as biofilm supports due to their capacity to enhance extracellular electron transfer in anaerobic wastewater treatment systems.^[^ ^5^ ^]^ Among various conductive materials, biochar provides a stable and biocompatible environment for electron transfer but has limited electron exchange ability, while iron‐based materials could promote electron transfer through excellent conductivity and redox properties.^[^ ^6^ ^]^ Owing to the synergistic effect of the combination of biochar and iron‐based materials, iron‐carbon composites have emerged as preferred additives due to their excellent biocompatibility and strong specific affinity for cytochrome c (CytC),^[^ ^1^, ^6^ ^]^ which are key components in microbial electron transport. However, these materials are typically introduced in the form of suspended powders, which limits their ability to support stable and structured biofilm development. Additionally, conventional commercial carriers (e.g., carbon felt, polyester sponges, carbon fiber brushes) often suffer from high interfacial electron transfer resistance, low surface wettability, and limited electrical conductivity, all of which hinder their effectiveness in facilitating electron and mass transfer.^[^ ^7^ ^]^ To overcome these limitations, it is essential to rationally design redox‐active conductive carriers that can simultaneously support robust biofilm formation, promote direct cell‐to‐cell interactions, and enhance interfacial electron transfer.

In anaerobic microbial communities, direct interspecies electron transfer (DIET) has been well‐established as a fundamental syntrophic mechanism among microbial species,^[^ ^8^ ^]^ where electrons are exchanged through conductive pili (e‐pili) or CytC between syntrophic partners. In contrast, direct electron transfer (DET) refers to a direct interfacial electron acceptance by methanogens from solid‐phase conductive materials via redox active substances (e.g., e‐pili, CytC),^[^ ^9^ ^]^ remains poorly understood, particularly in mixed microbial cultures. While some studies have demonstrated DET from elemental iron to certain methanogens in pure cultures (e.g., marine lithoautotrophic *Methanobacterium*‐like archaea),^[^ ^10^, ^11^ ^]^ conclusive evidence in mixed microbial systems is scarce. Crucially, both DIET and DET depend on the presence of key biological structures, such as e‐pili or CytC, to facilitate electron flow.^[^ ^12^ ^]^ Notably, DET can be influenced by the redox potential of carrier surfaces.^[^ ^13^ ^]^ Recent studies suggested that carriers equipped with surface‐bound redox‐active groups may lower energy barriers and thermodynamically drive electron transfer.^[^ ^14^, ^15^ ^]^ Despite this, the regulatory mechanisms of DET in biofilm and its coexistence with DIET remain largely unexplored in engineered anaerobic systems.

**Figure 1:**
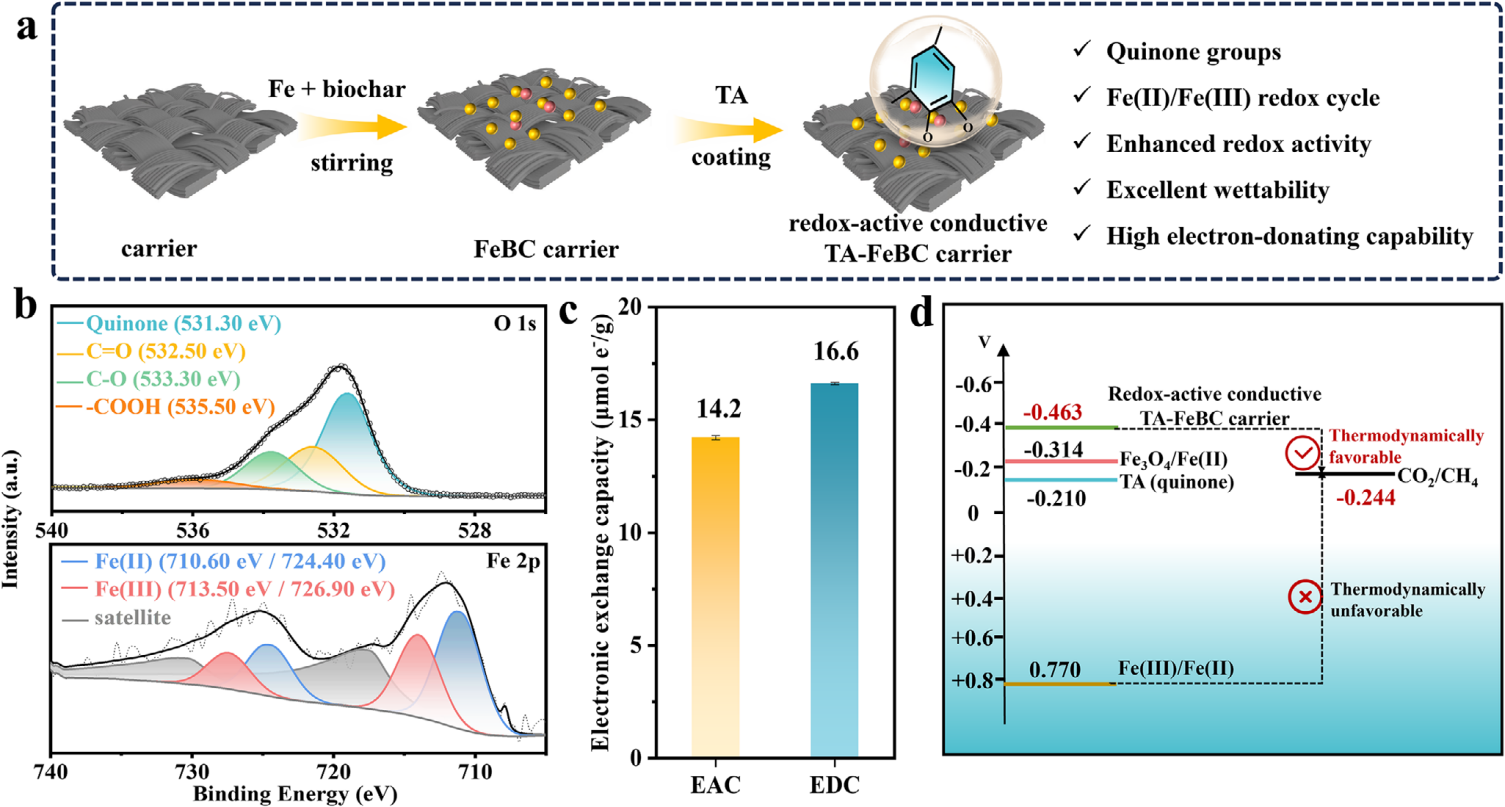
Enhanced redox activity and electron‐donating capacities of redox‐active conductive TA‐FeBC carrier. a) Schematic illustration of the synthesis process of the redox‐active conductive TA‐FeBC carrier; b) XPS spectra of O 1s and Fe 2p regions for the redox‐active conductive TA‐FeBC carrier; c) Electron‐accepting and donating capacities (EAC/EDC) of the redox‐active conductive TA‐FeBC carrier; d) Schematic illustration of the redox potentials of the redox‐active conductive TA‐FeBC carrier enabling DET with reference to the standard hydrogen electrode (SHE) at circumneutral pH.

Given this knowledge gap, we propose that anaerobic biofilm formed on the redox‐active conductive carriers with tailored redox potentials can facilitate DET at the anaerobic biofilm‐carrier interface through bacteria‐associated CytC and e‐pili. Herein, we designed a redox‐active conductive carrier by in situ fabrication of tannic acid‐modified iron‐biochar on carbon felt (i.e., the redox‐active conductive TA‐FeBC carrier) (**Figure** **1**a), which established a favourable redox‐active interface to facilitate direct electron transfer within anaerobic biofilm. The quinone groups present in tannic acid serve as key active sites for electron acceptance and transfer, effectively lowering the redox potential of the Fe(II)/Fe(III) pair while promoting the Fe(II)/Fe(III) redox cycle. Comprehensive electrochemical characterization and related analyses confirmed a thermodynamically driven mechanism based on redox potential, demonstrating direct transfer of electrons supplied by the redox‐active conductive TA‐FeBC carrier to anaerobic biofilm. Multiple analytical techniques and biochemical assays were employed to visualize biofilm metabolic activity and to elucidate the CytC‐mediated molecular mechanisms underlying DET. We therefore propose that DET was facilitated by CytC‐mediated pathways on the redox‐active conductive carrier biofilm, while DIET occurred concurrently among functionally synergistic microbial species. Metatranscriptomic analysis revealed upregulation of DET‐ and DIET‐related genes, highlighting metabolic reprogramming in response to the redox‐active interface on the conductive TA‐FeBC carrier. Consequently, this study provides experimental validation that the redox‐active conductive TA‐FeBC carrier can serve as an electron donor in anaerobic biofilm. By elucidating the molecular and thermodynamic basis of DET processes from the redox‐active conductive TA‐FeBC carrier, our findings could offer new insights into the design principles of redox‐active conductive carriers and expand the mechanistic understanding of DET in engineered anaerobic systems.

## 2 Results

### 2.1 Electron‐Donating Capability of Redox‐Active Conductive TA‐FeBC Carrier

#### 2.1.1 Surface Wettability of Redox‐Active Conductive TA‐FeBC Carrier

The wetting properties of a carrier can significantly influence the interfacial electronic interactions between microbes and the material, as well as affect mass transfer and reaction kinetics.^[^ ^16^ ^]^ Despite growing interest in the conductive carrier, wettability remains an underprioritized design variable in many studies. This study showed that aqueous solutions could not infiltrate the pristine carrier (i.e., carbon felt), thereby impeding both convective and diffusive mass transport between the carrier surface and the surrounding medium. In contrast, the redox‐active conductive TA‐FeBC carrier exhibited rapid and sustained surface wettability (Movie S1, Supporting Information). Specifically, its water contact angle decreased markedly from 152.3 ± 3.0° (hydrophobic) for the unmodified carrier to 15.0 ± 0.4° (hydrophilic) for the redox‐active conductive TA‐FeBC carrier (Figure S1, Supporting Information), highlighting the role of oxygen‐containing functional groups of TA in enhancing surface wettability. It should be noted that wettability governs critical microscale interactions at the anaerobic biofilm‐carrier interface, influencing not only microbial adhesion but also the spatial organization and functional expression of redox‐active proteins and e‐pili. On the other hand, DET requires tight and stable contact between solid‐phase electron donors (e.g., conductive carrier) and microbial redox structures as electron acceptors. This interfacial coupling is not solely dependent on electrical conductivity or the specific surface area of the carrier. Instead, it is essentially determined by the physicochemical characteristics of the interface, particularly surface wettability plays a critical role in shaping electron transfer. The excellent wettability of the redox‐active conductive TA‐FeBC carrier is expected to overcome the substantial resistance imposed by the “gating effect” of conventional commercial carriers with a hydrophobic nature. These indicate that surface wettability emerges as a key factor governing DET, while enhancing surface wettability is essential for promoting DET in anaerobic biofilm. Therefore, the systematic integration of wettability control into the design of conductive carriers is essential to fully unlock their potential in facilitating DET.

#### 2.1.2 Fe(II)/Fe(III) and Quinone‐Driven Redox Cycling

The anaerobic biofilm was developed on the redox‐active conductive TA‐FeBC carrier with the interlaced fibrous networks of ≈5 µm diameter, favorable for microbial attachment. The Fourier transform infrared spectroscopy (FTIR) spectra showed that narrow and strong peaks at 1580 and 1620 cm^−1^ were associated with quinoid C = O stretching vibration (Figure S2, Supporting Information),^[^ ^17^ ^]^ which was linked to redox characteristics of the redox‐active conductive TA‐FeBC carrier. The peak at ≈500 cm^−1^ could be assigned to the stretching and vibrational modes of the Fe─O bond.^[^ ^18^ ^]^ Furthermore, the characteristic peak of the aliphatic group of TA at 1120–1200 cm^−1^ was exclusively present in the redox‐active conductive TA‐FeBC carrier, where quinone moieties were effectively linked to iron through the C─O─Fe coordination bond, accelerating the conductive network formation.^[^ ^19^ ^]^ The high‐resolution spectrum of O 1s in Figure 1b was well divided into four peaks with binding energies of 531.3, 532.5, 533.3, and 535.5 eV, corresponding to quinone, C = O, C‐O, and ‐COOH,^[^ ^20^ ^]^ while the X‐ray photoelectron spectroscopy (XPS) analysis further revealed that the characteristic peaks located at 710.6 and 713.5 eV, corresponding to the deconvoluted Fe 2p_3/2_ spectra of Fe(II) and Fe(III), respectively (Figure 1b), with a calculated Fe(II)/Fe(III) ratio of 1.28. These together indicated the occurrence of a quinone‐mediated Fe(II)/Fe(III) redox cycle on the redox‐active conductive TA‐FeBC carrier, creating a redox cycling platform to facilitate electron transfer during anaerobic processes.

#### 2.1.3 Enhanced Redox Activity of Redox‐Active Conductive TA‐FeBC Carrier

The electron accepting capacity (EAC) and electron donating capacity (EDC) of redox‐active conductive TA‐FeBC carrier were determined to be 14.2 and 16.6 µmol e^−1^ g^−1^, respectively (Figure 1c), which were over 5‐fold higher than those reported for conventional conductive carriers (e.g., carbon felt, magnetite‐contained biochar, polymer‐modified activated carbon carrier, etc).^[^ ^21^, ^22^, ^23^ ^]^ In fact, EAC and EDC can directly reflect the amount of oxidizing and reducing substances available for electron transfer.^[^ ^24^, ^25^ ^]^ The significant increase in EDC suggests that TA as an iron coordination site as well as a reductive ligand facilitates electron transfer from quinone to Fe (III), thereby enhancing the redox activity of Fe(III) and Fe(II) on the conductive TA‐FeBC carrier. It was also found in Electrochemical impedance spectra (EIS) measurements that the electron transfer resistance (R_ct_) of optimally redox‐active conductive TA‐FeBC carrier with Fe:TA molar ratio of 5:3 was approximately one order of magnitude lower than that of commercial carbon felt (Figure S3, Supporting Information). Thus, it is expected that the electron transfer resistance at the carrier‐biofilm interface can be substantially lowered, creating a favorable environment for direct electron transfer between the redox‐active conductive TA‐FeBC carrier and the anaerobic biofilm.

Cyclic voltammetry (CV) measurements (Figure S4, Supporting Information) also showed that redox‐active conductive TA‐FeBC carrier had prominent oxidation and reduction peaks at −0.596 V (vs Ag/AgCl) and −0.724 V (vs Ag/AgCl), with the redox potential sufficiently lower than that of CO_2_/CH_4_. These offered thermodynamic insights into the ability of the redox‐active conductive TA‐FeBC carrier in direct electron transfer (Figure 1d). Additionally, the area obtained through integrating the CV curve showed that the capacitance of redox‐active conductive TA‐FeBC carrier reached 397 µF cm^−^^2^, which was over 10 times higher than that of other state‐of‐the‐art conductive materials (e.g., graphene, MXenes),^[^ ^26^, ^27^ ^]^ indicating a higher electron storage capacity of redox‐active conductive TA‐FeBC carrier, which could effectively boost a fast electron transfer at the carrier‐biofilm interface. Therefore, the quinone groups‐mediated iron redox cycle enabled the creation of an excellent redox‐active while conductive microenvironment, i.e., the redox‐active conductive TA‐FeBC carrier had more negative redox centers that promoted DET from the carrier to the anaerobic biofilm due to the lower potential barrier.

### 2.2 DET Enabled by Redox‐Active Conductive TA‐FeBC Carrier in Anaerobic Biofilm

#### 2.2.1 Enhanced Performance of Anaerobic Biofilm

**Figure 2:**
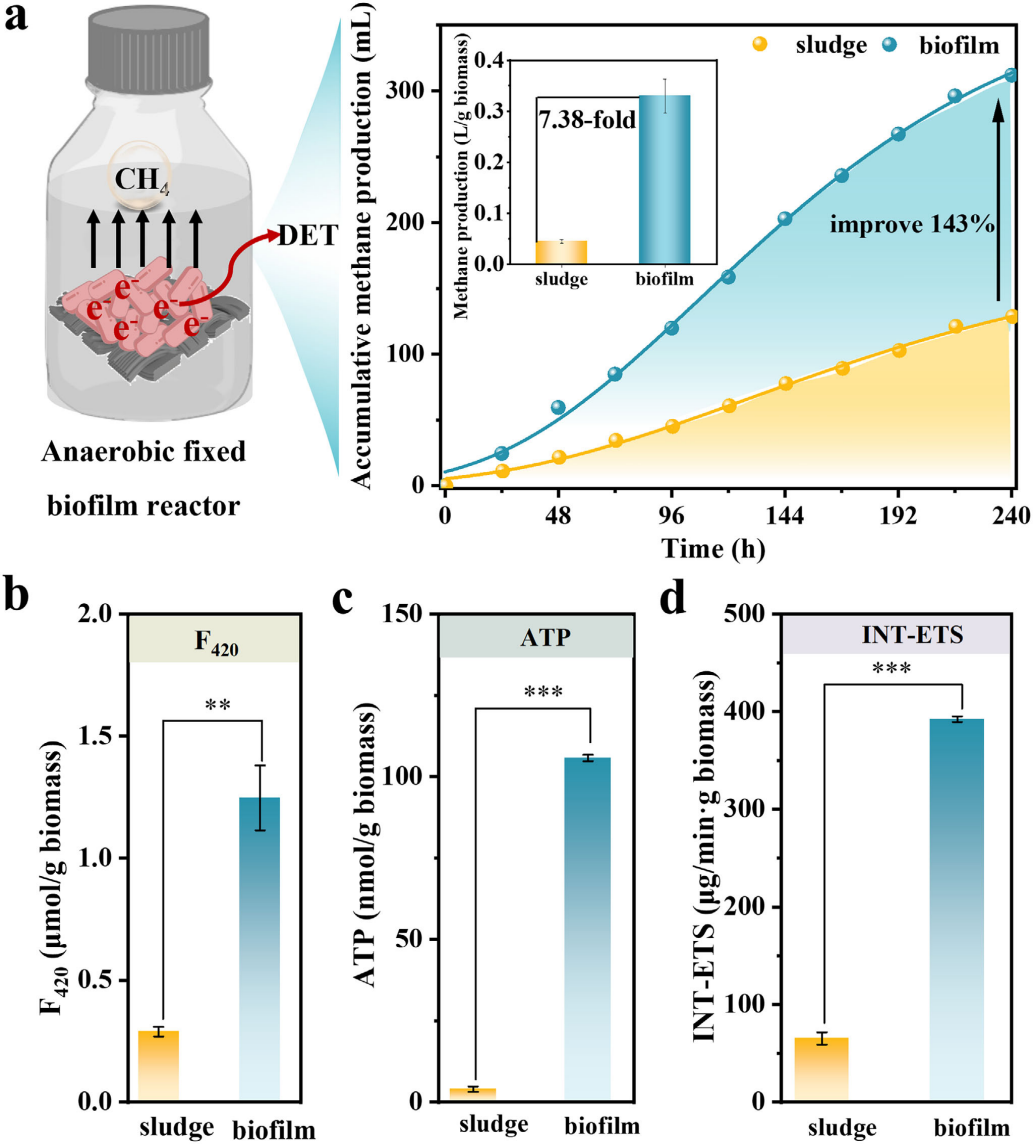
Performance of methanogenesis and key enzyme activity with the supplementation of the redox‐active conductive TA‐FeBC carrier via DET. a) The schematic illustration of methanogenesis facilitated by the redox‐active conductive TA‐FeBC carrier via DET, along with accumulative methane production in the control and the redox‐active conductive TA‐FeBC carrier reactors (inset: methane yield normalized to biomass in L g^−1^ for anaerobic suspended sludge and anaerobic biofilm); b) Changes in coenzyme F_420_ activity; c) ATP concentrations and d) INT‐ETS activities in anaerobic suspended sludge and anaerobic biofilm to quantify key enzymatic and electron transfer activities at the redox‐active conductive TA‐FeBC carrier‐biofilm interfaces. Sludge and biofilm in the figure refer to the anaerobic suspended sludge in the control group and the anaerobic biofilm on the redox‐active conductive TA‐FeBC carrier, respectively.

Anaerobic biofilm was developed on the redox‐active conductive TA‐FeBC carrier. The cumulative methane yield was increased by 143% for anaerobic biofilm on the redox‐active conductive TA‐FeBC carrier with a shorter lag phase as compared to the control group (**Figure** **2**a; Table S1, Supporting Information). A similar trend was also observed for total organic carbon (TOC) removal, e.g. 96.0 ± 2.7% versus 74.5 ± 10.7% in the control group (Figure S6, Supporting Information). Notably, the anaerobic biofilm on the redox‐active conductive TA‐FeBC carrier achieved a methane yield of 0.332 L g^−1^ biomass, which was 7.38 times higher than that of anaerobic suspended sludge (Figure 2a, inset). These findings together offered indirect evidence for the occurrence of DET at redox‐active conductive TA‐FeBC carrier‐biofilm interfaces, leading to enhanced methanogenic performance.

#### 2.2.2 Enhanced Electron Generation and Transfer at Redox‐Active Conductive TA‐FeBC Carrier‐Biofilm Interfaces

We determined coenzyme F_420_ in an anerobic biofilm, which was relevant to the CO_2_ reduction through directly receiving electrons from the chains of methanogens, and found that the coenzyme F_420_ content in anaerobic biofilm was 3.3 times higher than that of anaerobic suspended sludge (Figure 2b). These findings indicated a more effective transmembrane transfer of electrons while expediting anaerobic reduction of CO_2_ to CH_4_ at redox‐active conductive TA‐FeBC carrier‐biofilm interfaces. Moreover, the adenosine triphosphate (ATP) content of anaerobic biofilm (i.e., 105 nmol g^−1^ biomass) was 15.5 times higher than that of anaerobic suspended sludge (Figure 2c) as a result of the extracellular electron giving from the redox‐active conductive TA‐FeBC carrier to anaerobic biofilm. Together, these provided experimental evidence in support of direct electron transfer at the redox‐active conductive TA‐FeBC carrier‐anaerobic biofilm interfaces.

We further conducted intracellular electron transporting system (INT‐ETS) activity, EAC, and EDC tests to quantify internal electron generation activity and external electron‐accepting/donating capacity of anaerobic biofilm on the redox‐active conductive TA‐FeBC carrier. The INT‐ETS reflecting microbial respiration activity was used as an indicator of intracellular electron transfer rate. The INT‐ETS of anaerobic biofilm (e.g., 392 µg min^−1^ g^−1^ biomass) was 5.0 times higher than that of anaerobic suspended sludge (i.e., 65.4 µg min^−1^ g^−1^ biomass) (Figure 2d). Furthermore, the EAC and EDC values calculated from the electrochemical reduction and oxidation data were 4.50 and 5.14 µmol e^−1^ g^−1^ biomass for anaerobic biofilm against 0.524 and 0.322 µmol e^−1^ g^−1^ biomass for anaerobic suspended sludge (Figure S7, Supporting Information), evidencing a superior electron transfer activity of the redox‐active conductive TA‐FeBC carrier‐supported anaerobic biofilm. Consequently, the redox‐active microenvironment created between the anaerobic biofilm and the redox‐active conductive TA‐FeBC carrier enabled interfacial DET between bacteria and the carrier.

### 2.3 Electrochemical Measurements of DET in Anaerobic Biofilm

#### 2.3.1 Electron Transfer Behaviors at Redox‐Active Conductive TA‐FeBC Carrier‐Biofilm Interface

**Figure 3:**
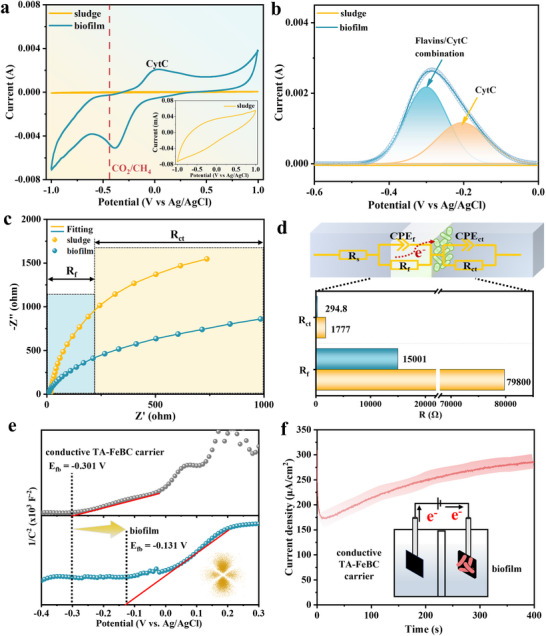
DET from the redox‐active conductive TA‐FeBC carrier to anaerobic biofilm was experimentally evidenced through comprehensive electrochemical characterization. Electrochemical properties of anaerobic biofilm probed by a) CV curves, b) the multi‐peak resolution results of DPV curves. c) EIS Nyquist plots and d) resistance fitted with an equivalent circuit. e) Mott‐Schottky plot of anaerobic biofilm in the absence or presence of the redox‐active conductive TA‐FeBC carrier at 200 Hz to obtain the flat‐band potential and the surface electron density. f) Cathodic current density of anaerobic biofilm, showing electron uptake from the redox‐active conductive TA‐FeBC carrier poised by anaerobic biofilm. Sludge and biofilm in the figure refer to the anaerobic suspended sludge in the control group and the anaerobic biofilm on the redox‐active conductive TA‐FeBC carrier, respectively.

We carried out the CV test to further investigate the electron transfer behavior at the redox‐active conductive TA‐FeBC carrier‐anaerobic biofilm interface. It was observed in **Figure** **3**a that the electron‐storage capacity of anaerobic biofilm was found to be 10.2 times higher than that of anaerobic suspended sludge, manifesting that anaerobic biofilm possessed more redox‐active species to modulate electron transfer behavior. Different from anaerobic suspended sludge, the oxidation peak at 0.050 V (vs Ag/AgCl) in anaerobic biofilm agreed well with the reported potential of CytC,^[^ ^28^ ^]^ and this was also supported by the higher expression of CytC‐related genes in anaerobic biofilm (Figure 6). On the other hand, the reduction peak potential (−0.386 V vs Ag/AgCl) in anaerobic biofilm was coincided with the standard potential of CO_2_ directly obtains electrons for CH_4_ production.^[^ ^29^ ^]^ Consequently, these supported the DET from the redox‐active conductive TA‐FeBC carrier to methanogens in the anaerobic biofilm.

To gain more insights into DET between the redox‐active conductive TA‐FeBC carrier and anaerobic biofilm, we determined the number of associated electroactive substances by using differential pulse voltammetry (DPV). As shown in Figure 3b, two obvious oxidative peaks of anaerobic biofilm were observed in the DPV curves at −0.198 V (vs Ag/AgCl) and −0.300 V (vs Ag/AgCl), which represented the CytC and the flavins‐CytC complex, respectively. Both have been known to regulate the interaction with extracellular electron donors,^[^ ^30^ ^]^ whereas flavins secreted by microbial cells could participate in DET as a cofactor for CytC.^[^ ^31^ ^]^ Furthermore, the total number of associated electroactive substances on DET sites was calculated in terms of the formal charge (Q_formal_) obtained from the DPV peak area. The Q_formal_ values were found to be 8.87 × 10^−2^ C and 1.39 × 10^−1^ C for the CytC and flavins‐CytC complex in the anaerobic biofilm (Table S2, Supporting Information), suggesting that the anaerobic biofilm could promote direct interaction of CytC with the redox‐active conductive TA‐FeBC carrier while facilitating DET at the anaerobic biofilm‐carrier interface.

#### 2.3.2 Electron Transfer Resistance at Redox‐Active Conductive TA‐FeBC Carrier‐Biofilm Interfaces

The electron transfer kinetics at the redox‐active conductive TA‐FeBC carrier‐anaerobic biofilm interface was studied by EIS measurements (Figure 3c) with which the resistance of the anaerobic biofilm adhering to the redox‐active conductive TA‐FeBC carrier (R_f_), and the electron transfer resistance (R_ct_) between the anaerobic biofilm and the redox‐active conductive TA‐FeBC carrier were determined from equivalent circuit models. It was shown that the R_f_ of anaerobic biofilm was almost 4.33 times lower than that of anaerobic suspended sludge, indicating the presence of more redox substances in the redox‐active conductive TA‐FeBC carrier‐supported anaerobic biofilm while accelerating the bioelectrochemical reactions. As a predominant restrictive factor for electron transfer, R_ct_ of anaerobic biofilm was 50.9‐fold lower than that of anaerobic suspended sludge (Figure 3d). It is evident that the lower R_ct_ is essential for creating an ideal highway for DET in the redox‐active conductive TA‐FeBC carrier‐supported anaerobic biofilm.

#### 2.3.3 Electrochemical Measurement of DET from Redox‐Active Conductive TA‐FeBC Carrier to Biofilm

To experimentally demonstrate the DET, we constructed typical Mott–Schottky plots for the redox‐active conductive TA‐FeBC carrier in the presence and absence of anaerobic biofilm (Figure 3e). The flat‐band potentials (E_fb_) obtained for anaerobic biofilm and the redox‐active conductive TA‐FeBC carrier were respectively determined to be −0.131 V (vs Ag/AgCl) and −0.301 V (vs Ag/AgCl), indicating that the formation of anaerobic biofilm on the redox‐active conductive TA‐FeBC carrier led to a bending of energy bands, ultimately making the E_fb_ position shifted upward to match the energy level of CytC. In addition, the surface electron density for the anaerobic biofilm was (i.e., 14.4) was 5 times greater than that of the redox‐active conductive TA‐FeBC carrier (i.e., 2.85). Such a larger difference in the surface electron density between the anaerobic biofilm and the redox‐active conductive TA‐FeBC carrier could create a redox gradient, allowing the fast and effective electron transfer from the redox‐active conductive TA‐FeBC carrier to the anaerobic biofilm. On the other hand, the E_fb_ of redox‐active conductive TA‐FeBC carrier was higher than the redox potential of CytC, but lower than that of the Fe(II)/Fe(III), indicating that the electrons were spontaneously transferred from the conduction band of the redox‐active conductive TA‐FeBC carrier to CytC of anaerobic biofilm. As the result, the built‐in electric field generated by the potential difference between anaerobic biofilm‐associated CytC and the Fe(II)/Fe(III) redox pair on the redox‐active conductive TA‐FeBC carrier can effectively promote DET at the anaerobic biofilm‐carrier interface. To further experimentally evidence DET, we also designed the experiments with the redox‐active conductive TA‐FeBC carrier as a simulated electron source to measure the current flow between methanogens in the anaerobic biofilm and the redox‐active conductive TA‐FeBC carrier. It was found that methanogens in the anaerobic biofilm can directly accept the electrons released from the redox‐active conductive TA‐FeBC carrier while generating a strong cathodic current of 110 µA cm^−2^ (Figure 3f). These offer experimental evidence in support of DET in the anaerobic biofilm on the redox‐active conductive TA‐FeBC carrier.

### 2.4 Molecular Mechanisms of DET in Anaerobic Biofilm

**Figure 4:**
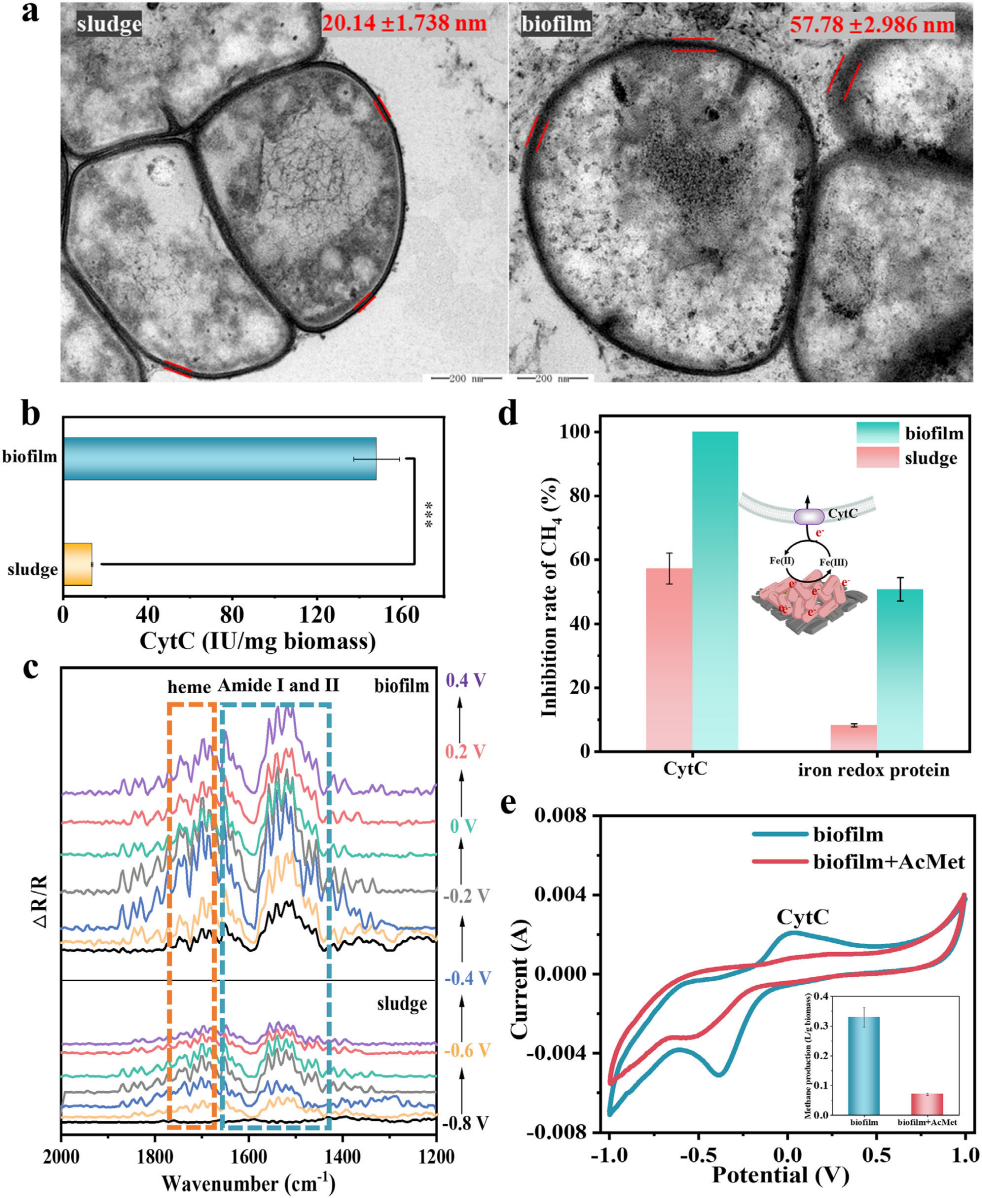
Molecular mechanisms of CytC‐mediated transmembrane DET were proved by TEM visualization, in situ spectroscopic analyses, and electron transport inhibitors. a) TEM images with DAB‐H_2_O_2_ staining for the distribution of CytC on cell membranes, b) CytC concentrations in anaerobic suspended sludge and anaerobic biofilm, and c) electrochemical in situ FTIR spectra to detect CytC of anaerobic suspended sludge and anaerobic biofilm at the molecular level; d) Effects of extracellular electron transfer inhibitors on CH_4_ production. e) The changes on CV curves and methane production (inset) in the protein‐unfolding experiments with the supplementation of acetyl methionine (AcMet, ligand coordination reaction with CytC) in anaerobic biofilm.

To substantiate the role of CytC, a key electron transfer structural protein CytC in DET‐driven activity, we quantified and visualized CytC in the anaerobic biofilm on the redox‐active conductive TA‐FeBC carrier. Transmission electron microscopy (TEM) with 3,3′‐diaminobenzidine (DAB) and H_2_O_2_ staining revealed that the CytC ring in the extracellular membrane of the anaerobic biofilm (57.8 ± 3.0 nm) was ≈2.9 time thicker than that of anaerobic suspended sludge (20.1 ± 1.7 nm) (**Figure** **4**a), while no staining was visible for cells treated with DAB alone for both anaerobic biofilm and suspended sludge (Figure S8, Supporting Information). Meanwhile, the CytC content in the anerobic biofilm was ≈10.8 times higher than that in anaerobic suspended sludge (Figure 4b). These indicate that the anaerobic biofilm had more extracellular‐to‐intracellular conductive channels allowing more efficient transmembrane electron transfer.

To further confirm the CytC‐mediated DET at anaerobic biofilm‐carrier interface, we recorded the electrochemical in situ FTIR spectra of CytC at the molecular level for both anaerobic biofilm and suspended sludge. According to the CV curves in Figure 3a, the potential was set to the range of −0.8 to 0.4 V during the collection of the FTIR spectra, allowing full oxidation and reduction of CytC. Significant changes of typical CytC vibrational bands as potential shifted from −0.8 to 0.4 V were observed for anaerobic biofilm, but not for anaerobic suspended sludge (Figure 4c). Specifically, the bands at 1710 and 1405 cm^−1^ respectively, represented the stretching and bending vibration of the heme group in the surface of CytC,^[^ ^8^ ^]^ showing the abundant presence of CytC in anaerobic biofilm, which was in good agreement with the findings of Figure 4a,b. On the other hand, the intensities of the bands at 1650 and 1550 cm^−1^ belonging to the amide I group of C = O and the amide II group of C‐N for anaerobic biofilm varied more significantly than those for anaerobic suspended sludge at the potential range of −0.8–0.4 V, indicating a potential‐induced polarization of the anaerobic biofilm.^[^ ^32^ ^]^ The highly polarized C‐N and C = O in amide groups reflected a high‐energy state of electrons in anaerobic biofilm,^[^ ^33^ ^]^ which likely favored the transmembrane DET. The electrochemical in situ FTIR analysis offers additional support to the CytC‐mediated DET in anaerobic biofilm on the redox‐active conductive TA‐FeBC carrier. Dimercaprol dimercaptopropanol (BAL) has been known to inhibit the electron transfer to CytC.^[^ ^34^ ^]^ Figure 4d showed that the methane production of anaerobic biofilm was completely inhibited in the presence of BAL due to the blockage of the electron transfer to CytC. Meanwhile, it was observed that the inhibited methane production by the inactivation of iron redox protein was alleviated, with the inhibition rate decreasing from 50.7% for anaerobic biofilm to 8.23% for anaerobic suspended sludge. These suggest that the Fe(II)/Fe(III) cycle could reinforce the CytC‐mediated DET in the redox‐active conductive TA‐FeBC carrier‐supported anaerobic biofilm. To gain more insights, we performed further protein‐unfolding experiments with acetyl methionine (AcMet) which undergoes the ligand coordination reaction with CytC. The addition of AcMet resulted in reduced methane production by 78.8% in anaerobic biofilm (Figure 4e, inset). This was likely due to the fact that AcMet could impede electron transfer by binding to the outer membrane CytC and thus creating a substantial energy barrier for electron transfer.^[^ ^35^ ^]^ The disappearance of the oxidation peak assigned to CytC and the negative shift of the reduction peak (−0.534 V vs Ag/AgCl) once again manifested the involvement of the anaerobic biofilm‐CytC in mediating transmembrane DET (Figure 4e).

### 2.5 Synergistic Direct and Interspecies Electron Transfer in Anaerobic Biofilm

**Figure 5:**
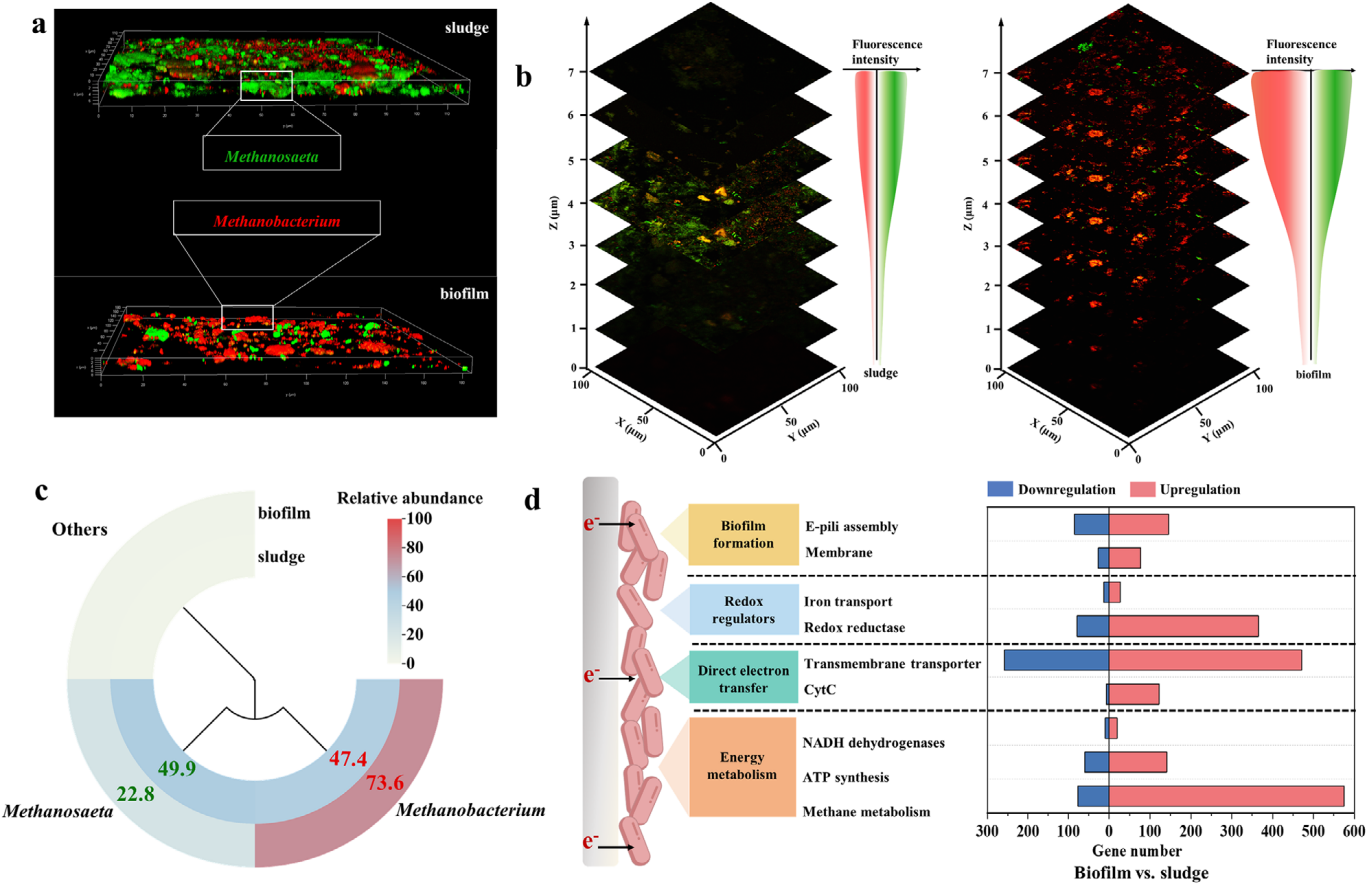
Microbial selective enrichment behaviors and DET‐related gene expression in anaerobic biofilm. a) 3D FISH‐CLSM images of anaerobic suspended sludge and anaerobic biofilm, *Methanobacterium* was targeted with a Cy3‐labeled probe (red), while *Methanosaeta* was targeted with a MX825‐FAM ‐labeled probe (green); b) Stacked confocal images at different heights Z for the distribution of methanogens in anaerobic suspended sludge and anaerobic biofilm; c) The community structure analysis of archaea at the genus level of anaerobic suspended sludge and anaerobic biofilm (genus with relative abundance below 1.0% categorized into others); d) Classification of self‐defined categories based on direct electron transfer in upregulated and downregulated genes (anaerobic biofilm on redox‐active conductive TA‐FeBC carrier vs anaerobic suspended sludge).

We performed the florescence in situ hybridization‐confocal laser scanning microscopy (FISH‐CLSM) imaging analysis to elucidate the synergy between the functional microorganisms in anaerobic biofilm and anaerobic suspended sludge. The anaerobic suspended sludge enriched *Methanosaeta*, while *Methanobacterium* was selectively enriched in the anaerobic biofilm (**Figure** **5**a). Figure 5b presents a detailed distribution of *Methanosaeta* and *Methanobacterium* across the vertical direction (Z) from the bottom to the top cross‐sections of anaerobic biofilm and anaerobic suspended sludge. Notably, *Methanobacterium* exhibited a higher average fluorescence intensity in the top layer of the anaerobic biofilm compared to the bottom layer, which may be attributed to the facilitation of electron transfer by the redox‐active conductive TA‐FeBC carrier. It has been reported that *Methanosaeta* participated in DIET,^[^ ^36^ ^]^ in contrast *Methanobacterium* is capable of utilizing electrons from solid abiotic materials for a highly selective production of methane.^[^ ^11^ ^]^ The relative abundance of *Methanosaeta* in the anaerobic suspended sludge was 49.9%, while *Methanobacterium* accounted for 73.6% in the anaerobic biofilm on the redox‐active conductive TA‐FeBC carrier (Figure 5c). From a microbiological point of view, these findings suggested that the symbiotic redox‐active conductive TA‐FeBC carrier‐anaerobic biofilm system enabled the synergetic DIET and DET at the anaerobic biofilm‐carrier interface, thereby promoting efficient electron transfer from carbon dioxide to methane.

We also analyzed the methanogenic metabolic pathways to gain insights into the synergetic DIET and DET in anaerobic biofilm based on the metatranscriptomic platform. Compared with the anaerobic suspended sludge, 21 290 genes were significantly up‐regulated (FC≥1.2) and 10 594 genes significantly down‐regulated (FC≤0.83) in the anaerobic biofilm (Figure S9, Supporting Information), reflecting highly active metabolic responses in the anaerobic biofilm, which contributed to the system performance and stability. To further identify genes involved in shared regulatory pathways, key functional genes related to biofilm formation, redox regulation, DET, and energy metabolism were extracted based on the annotation of differentially expressed genes (Figure 5d). Notably, the expression of 122 genes encoding CytC, which were essential for DET at the anaerobic biofilm‐carrier interface, was significantly upregulated. This suggests that CytC plays a central role in sustaining electron transport within the anaerobic biofilm matrix. Moreover, the highest number of upregulated genes (i.e., 575) was associated with methanogenic metabolism, indicating that methanogenic pathways were strongly activated in the anaerobic biofilm.

**Figure 6:**
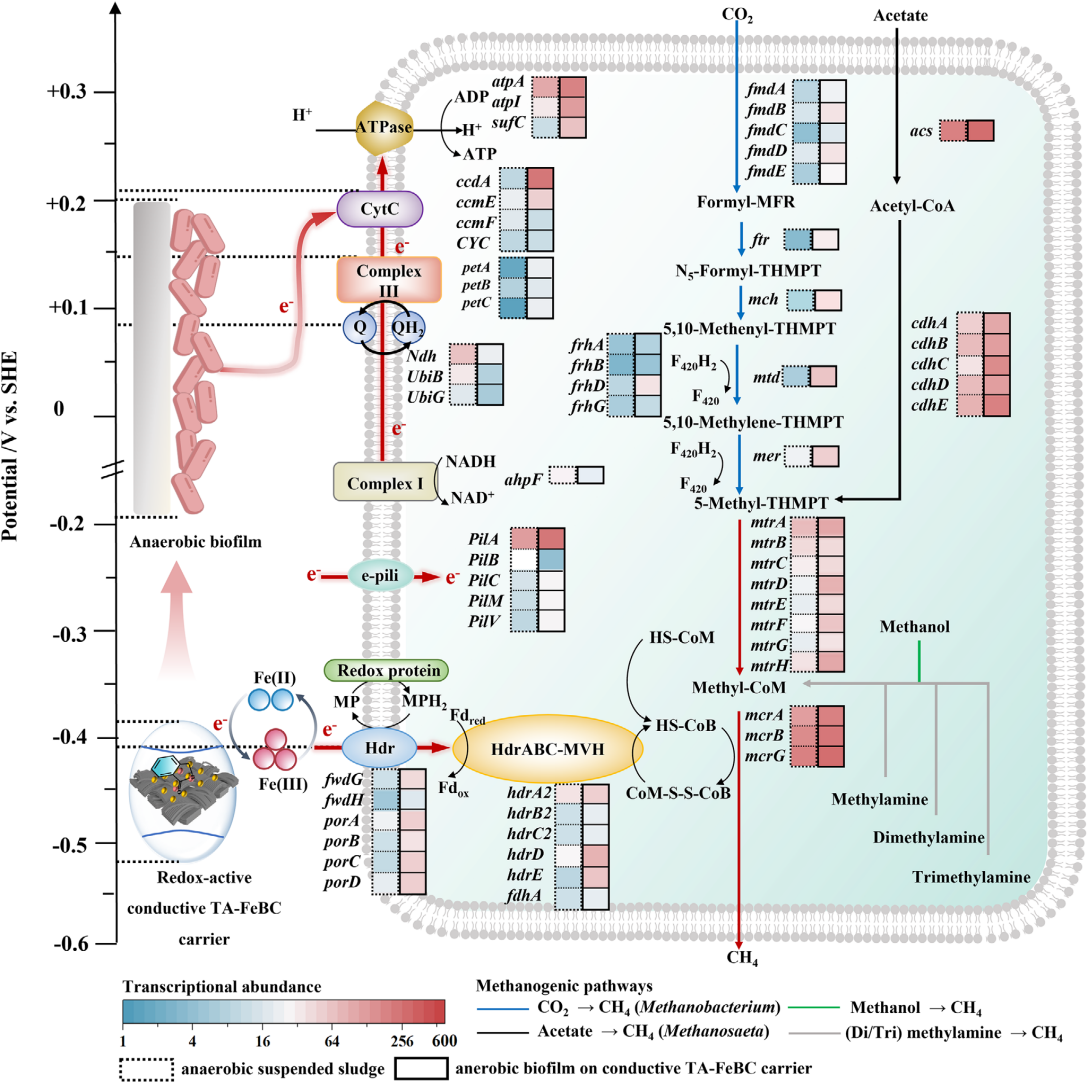
The construction of metabolic maps for the transcript abundance of key enzyme‐encoding genes in the methanogenic metabolism of anaerobic suspended sludge and anaerobic biofilm. The potentials on the left represent the redox potential values of redox‐active conductive TA‐FeBC carrier, anaerobic biofilm, and direct electron transfer components (e.g., CytC and iron redox protein (Fd_red_/Fd_ox_)) with reference to the standard hydrogen electrode (SHE) at circumneutral pH. The right section represents the transcript abundances of key genes related to methanogenic metabolism processes of anaerobic suspended sludge (black dashed square symbols) and anaerobic biofilm (black solid‐lined square symbols).

The CO_2_‐reducing methanogenesis (M00567) and acetoclastic methanogenesis (M00357) were further identified as the highly expressed units of methanogenesis in the anaerobic biofilm (Figure S10, Supporting Information), collectively contributing to the synergistic DET and DIET. Generally, *Methanosaeta* can convert acetate to acetyl‐CoA via acetyl‐CoA synthetase (*acs*) to accelerate DIET, which explains the high gene expression level of *acs* in the anaerobic biofilm for the acetoclastic methanogenesis module (Figure 6). Consequently, the redox‐active conductive TA‐FeBC carrier could enhance the expression of genes involved in CO_2_ reduction by leveraging its Fe(II)/Fe(III) redox pair and electron‐donating capability while creating favorable microenvironments for sustaining microbial growth and CO_2_ methanation. In addition, the relatively low redox potential of the redox‐active conductive TA‐FeBC carrier (−0.460 V vs SHE) enabled electron transfer to membrane‐bound ferredoxin (Fd_ox_/Fd_red_, −0.398 V vs SHE) via the Fe(II)/Fe(III) redox pair, making the redox‐active conductive TA‐FeBC carrier‐mediated DET thermodynamically feasible. The incorporation of Fe(II) and Fe(III) into cells via membrane‐bound protein containing cytochrome (*Hdr*), further enabled the transfer of electrons generated from the cycle of CoM‐S‐S‐CoB and HS‐CoB, driving the ATP synthesis and methanogenic DET.^[^ ^34^, ^37^ ^]^ It was found that *hdrA2*, *hdrB2*, *hdrC2*, *hdrD*, and *hdrE* in the anaerobic biofilm were also enriched by 1.44–12.0 times as compared to the anaerobic suspended sludge. On the other hand, efficient DET can be facilitated by enriched *Methanobacterium* at the anaerobic biofilm‐carrier interface (Figure 5b), which utilizes CO_2_ and electrons/protons to produce CH_4_ (−0.244 V vs SHE). Notably, the transcriptional abundance of formylmethanofuran dehydrogenase genes (*fmdABCDE*) responsible for the initiation of CO_2_ to CH_4_ conversion was 3.08–6.88 times higher in the anaerobic biofilm than in anaerobic suspended sludge. Moreover, the transcriptional abundance of *ccdA*, a key CytC gene was increased by 5.78 times in the anaerobic biofilm compared to the anaerobic suspended sludge (**Figure** **6**), while fostering contact‐based transmembrane DET at the anaerobic biofilm‐carrier interface with the CytC as the redox mediator.

We also explored the role of conductive pili (e‐pili) in the transmembrane electron transfer of the anaerobic biofilm. As the key e‐pili gene, *PilA* was found to be highly expressed in the anaerobic biofilm in comparison with anaerobic suspended sludge (Figure 6). The amounts of filamentous e‐pili formed by protein filaments in the anaerobic biofilm on the redox‐active conductive TA‐FeBC carrier reached 7.78 ng g^−1^ biomass, which was 7.40 times higher than that observed in the anaerobic suspended sludge (Figure S11, Supporting Information). It is apparent that the excellent conductivity of the redox‐active conductive TA‐FeBC carrier can regulate the expression of e‐pili, which further facilitates electron transfer from the redox‐active conductive TA‐FeBC carrier to methanogens. These together confirmed that the redox‐active conductive TA‐FeBC carrier creates multi‐dimensional conductive structures of the anaerobic biofilm which can effectively mediate DIET and DET.

## 3 Discussion

The exogenous incorporation of conductive carriers to construct anaerobic biofilm has emerged as a promising strategy to enhance extracellular electron transfer and improve methanogenic performance. Rational design of such carriers is critical for achieving more efficient and tightly coupled DET at the anaerobic biofilm‐carrier interface. For instance, the wettability of conductive carriers played a pivotal role in mediating interactions with anaerobic biofilms and served as a key variable for engineering advanced carriers in high‐performance anaerobic systems. Despite its importance in enabling effective DET at the anaerobic biofilm‐carrier interface, this critical factor is largely underappreciated. In this study, we showed that the redox‐active conductive TA‐FeBC carrier with excellent surface wettability facilitated both extracellular and transmembrane electron transfer at the microbial metabolic level, establishing a CytC‐mediated DET pathway from the carrier to methanogens. Electrochemical analyses verified the feasibility of this previously unreported electron transfer route, whereby electrons were directly extracted from the redox‐active conductive TA‐FeBC carrier and channeled into metabolic pathways.

**Figure 7:**
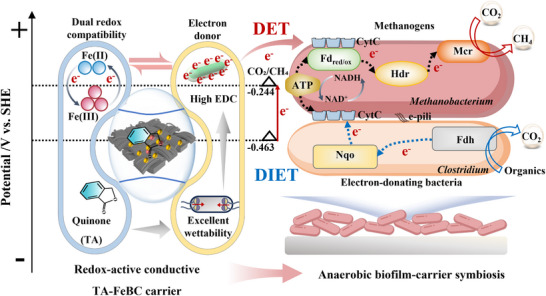
Schematic of redox‐active conductive TA‐FeBC biofilm driving dual‐pathway DET mechanism.

Importantly, the redox‐active conductive TA‐FeBC carrier exhibited dual redox compatibility with both the Fe(II)/Fe(III) redox couple and CytC, enabling it to function as a redox‐active interface that simultaneously supports both DET and DIET. This dual electron transfer capability, as illustrated in **Figure** **7**, highlights the formation of a biofilm‐carrier symbiosis capable of bidirectional electron mediation. The suitable redox potential of the TA‐FeBC carrier, which was lower than that of the CO_2_/CH_4_ couple, could effectively overcome the intrinsic energetic barriers associated with electron transport across cell membranes, thereby facilitating the seamless incorporation of electrons into cellular metabolic pathways.

Compared to conventional non‐modified conductive carriers, the redox‐active conductive TA‐FeBC carrier uniquely combines electroactivity and redox mediation, synergistically enhancing biofilm integrity, electron transfer efficiency, and metabolic activity. These features marked a significant advancement in conductive carrier engineering. The redox‐active conductive TA‐FeBC carrier‐enabled dual DET pathway offered the potential to spatially reorganize electron flow within anaerobic biofilm, providing a robust abiotic‐biotic interface for optimized electron distribution. In this study, we established a reproducible model for conductive carrier‐biofilm systems, which laid a foundation for extending this strategy across diverse environmental applications to support more sustainable and efficient anaerobic wastewater treatment. Future study will focus on developing novel carrier modules for long‐term operation in continuous‐flow anaerobic fixed biofilm reactors, thereby opening new avenues for large‐scale practical applications in wastewater treatment.

## 4 Experimental Section

### Synthesis of Redox‐Active Conductive TA‐FeBC Carrier and Electron Transfer Ability Analysis

The redox‐active conductive TA‐FeBC carrier was in situ fabricated via a hydrothermal method (Text S1, Supporting Information). The morphology and element distribution were imaged by field emission scanning electron microscopy (FESEM, JSM‐7800F) and EDS mapping (Figure S5, Supporting Information). Fourier transform infrared spectroscopy (FTIR, Bruker Tensor 27, Germany) was utilized to identify functional groups of carriers in the range of 4000–400 cm^−1^. The elemental composition and their valence states were obtained by X‐ray photoelectron spectroscopy (XPS, ESCALAB 250, Thermo Fisher Scientific). All binding energies were calibrated and referenced to the C 1s peak at 284.80 eV. The surface wettability of the redox‐active conductive TA‐FeBC carrier was evaluated using a contact angle goniometer (Kruss DSA23S, Germany) with 2 µL water droplets. All contact angle data were obtained at a contact time of 5 s, and calculated the average of three measurements. Electrochemical analysis was recorded on an electrochemical workstation (CHI760E, Chenhua, China) with a standard three‐electrode system. The redox‐active conductive TA‐FeBC carrier, platinum electrode, and Ag/AgCl electrode (+0.205 V vs standard hydrogen electrode, SHE) were adopted as the working, counter, and reference electrodes, respectively. Electrochemical impedance spectra (EIS) were performed over the frequency range of 0.1–10^5^ Hz, and the curves were handled by constructing equivalent circuit diagrams to fit the charge transfer resistance. The cyclic voltammetry (CV) curve was swept between −1.0 and 1.0 V at a rate of 50 mV s^−1^ to calculate the capacitance. The electron accepting capacity (EAC) and electron donating capacity (EDC) were tested in accordance with previous reports.^[^ ^38^ ^]^ The pretreated procedure of the pristine carrier (carbon felt) used in this study is shown in Text S2 (Supporting Information).

### Cultivation of Anaerobic Biofilm on Redox‐Active Conductive TA‐FeBC Carrier

To evaluate the impact of the synthesized redox‐active conductive TA‐FeBC carrier on biofilm formation, direct electron transfer, and microbial metabolism in anaerobic systems, enrichment experiments were conducted using the redox‐active conductive TA‐FeBC carrier. Anaerobic suspended sludge in the absence of a conductive carrier group was utilized as the control for comparison. Both the sludge and biofilm groups were inoculated with anaerobic sludge at a concentration of 5 g VSS/L, whereas the biofilm group was supplemented with a redox‐active conductive TA‐FeBC carrier connected to a vertically immersed Ti wire within the sludge suspension. The total projected area of the carrier was 8 cm^2^, and a loading density of conductive material was 1 mg cm^−2^ on the carbon felt. The experiment was conducted to cultivate anaerobic biofilm on redox‐active conductive TA‐FeBC carrier by semi‐continuous feeding mode for 20 operation cycles, and synthetic municipal wastewater was used as the substrate. More details on reactor setup, operating parameters, and performance analysis were provided in (Text S3, Supporting Information).

### Determinations of Key Enzymatic and Electron Transfer Activities

The activity of the coenzyme F_420_ was measured using the ultraviolet spectrophotometry method.^[^ ^39^ ^]^ The adenosine triphosphate (ATP) concentration was determined using an ATP assay kit (Beyotime Biotechnology Co., Ltd., Shanghai, China). The activity of electron transfer system (ETS) was determined by 2‐(p‐iodophenyl)‐3‐(p‐nitrophenyl)‐5‐phenyltetrazolium chloride (INT) ‐ETS method.^[^ ^40^ ^]^

### Electrochemical Analysis of Direct Electron Transfer in Anaerobic Biofilm

The redox activity and resistivity of the anaerobic biofilm were analyzed in a three‐electrode system by CV and electrochemical impedance spectroscopy (EIS) method respectively. The electron accepting capacity (EAC) and electron donating capacity (EDC) of anaerobic biofilm were characterized by mediated electrochemical reduction (MER) and oxidation (MEO), respectively.^[^ ^41^ ^]^ The differential pulse voltammetry (DPV) was performed to explore the effects of anaerobic biofilm on extracellular electric activity.^[^ ^30^ ^]^ The Mott–Schottky plot was obtained to explore the effects of anaerobic biofilm on the flat band of redox‐active conductive TA‐FeBC carrier. Electrochemical measurements were conducted in freshly prepared N_2_‐purged 0.1 m PBS (pH 7.4). The details of experimental procedure for confirming the hypothesis of direct electron transfer (DET) from the redox‐active conductive TA‐FeBC carrier to anaerobic biofilm was shown in (Text S4, Supporting Information).

### Microscopic Analysis of Biofilm

The morphology and distribution of functional microbial communities (*Methanosaeta* and *Methanobaterium*) interacting with anaerobic biofilm and anaerobic suspended sludge were visualized by Florescence in situ hybridization imaging with confocal laser scanning microscopy (FISH‐CLSM, Leica STELLARIS 5, Germany). Detailed information was shown in (Text S5, Supporting Information).

For transmission electron microscopy (TEM) analysis of anaerobic biofilm and anaerobic suspended sludge, the samples were washed with 0.01 m PBS (pH 7.4) and harvested by centrifugation at 5000 g for 5 min, immediately fixed in 2.5% glutaraldehyde prepared in 50 mm HEPES (pH 7.4), and stored overnight at 4 °C. A cytochrome‐reactive staining solution was prepared using a literature method and consisted of 1.5 g L^−1^ 3,3′‐diaminobenzidine tetrahydrochloride (DAB) and 0.02% H_2_O_2_ in 50 mm Tris‐HCl buffer solution (pH 8).^[^ ^42^ ^]^ A DAB solution without H_2_O_2_ addition was also prepared as the negative staining control. The staining solutions were applied to the embedded sections of the samples and incubated for 2 h at room temperature. The thin sections were deposited on 200 mesh carbon‐coated copper grids and observed by using TEM.

### Analysis of CytC‐Mediated DET at Anaerobic Biofilm‐Carrier Interface

The cytochrome c (CytC) concentration of anaerobic biofilm and anaerobic suspended sludge was tested by ELISA kits (Feiya Biotechnology Co., Ltd., Jiangsu, China). Electrochemical in situ FTIR spectroscopy was performed on a WQF‐530A FTIR spectrometer (Beijing Beifen‐Ruili Analytical Instrument(Group) Co., Ltd.) equipped with a liquid nitrogen‐cooled narrow‐band MCT‐A detector and a VeeMAX III ATR accessory (Pike Technologies) to identify CytC in anaerobic biofilm. The reference potential (ER) was set as −0.9 V, and the applied potentials were set in a region from −0.8 to 0.4 V with an interval of 0.2 V as previously described. All of these tests were conducted in a N_2_‐purged fresh 0.1 m PBS solution (pH 7.4).

To further substantiate the role of CytC in mediating DET at the anaerobic biofilm‐carrier interface, a protein‐unfolding assay was performed using 10 mm acetyl methionine (AcMet).^[^ ^35^ ^]^ Methane production was measured in the presence and absence of AcMet to assess the role of CytC in DET at the anaerobic biofilm‐carrier interface. CV was conducted to evaluate redox‐active species and verify the role of CytC in biofilm electron transfer processes. Following the 20‐cycle enrichment, the grown biofilm on redox‐active conductive TA‐FeBC carrier was distributed into four 150 mL anaerobic reactors (each with a 100 mL working volume) for inhibitor experiments. Using two metabolic inhibitors, BAL (targeting CytC) and CuCl_2_ (targeting the iron redox protein), to verify the roles of biofilm in influencing the transmembrane respiratory chain.

### Analysis of Microbial Community and Metabolic Pathway Shift of Biofilm

At the end of each experiment, 10 mL of anaerobic suspended sludge and 1 cm × 1 cm × 0.2 cm anaerobic biofilm samples were collected for microbial community and metatranscriptomic sequencing analysis. Detailed information was displayed in (Text S6, Supporting Information). The sequencing raw data were submitted to the National Center for Biotechnology Information (NCBI) Sequence Read Archive (http://www.ncbi.nlm.nih.gov/sra) with accession numbers SRR34705499 and SRR34705500 under the BioProject PRJNA1293658.

### Statistics Analysis

All experiments were conducted in triplicate, and the results are shown as mean ± standard deviation. For data analysis, one‐way analysis of variance (ANOVA) was applied to analyze statistical differences in the experimental data using Origin 2021 with ^*^ *p* < 0.05, ^**^ *p* < 0.01, and ^***^ *p* < 0.001.

## Conflict of Interest

The authors declare no conflict of interest.

## Author Contributions

J.T. performed the molecular synthesis, conducted the experiments, carried out the data analysis, and contributed to the original draft. X.Z. contributed to the original draft, performed data analysis, and provided supervision as well as manuscript writing and revision. L.X. carried out data analysis. B.J. contributed to manuscript revision. J.L. participated in manuscript writing and revision. Y.L. conceived and designed the experiments, provided supervision and conceptualization, secured funding, and contributed to manuscript writing and revision. All authors discussed and commented on the manuscript.

## Supporting information



Supporting Information

Supplementary Movie 1

## Data Availability

The data that support the findings of this study are available from the corresponding author upon reasonable request.
