# Supplementary material for: Mechanisms of Direct Electron Transfer Governed by Redox‐Active Conductive Carrier with Superior Wettability in Anaerobic Biofilms
Source: Adv Sci (Weinh). 2025 Nov 29;13(8):e16258. doi: 10.1002/advs.202516258 (PMC12884730; doi:10.1002/advs.202516258)
Supplement: Supplementary file 1 — Supporting Information [file ADVS-13-e16258-s002.docx]

**Supporting information**

**Mechanisms of Direct Electron Transfer Governed by Redox-Active Conductive Carrier with Superior Wettability in Anaerobic Biofilms**

Junli Tian^1^, Xiaoyuan Zhang^1,2*^, Lulu Xing^1^, Bin Ji^3^, Jinfeng Lu^1,2*^ & Yu Liu^1,2*^

^1^Engineering Laboratory of Low-Carbon Unconventional Water Resources Utilization and Water Quality Assurance, College of Environmental Science and Engineering, Nankai University, Tianjin 300350, China

^2^Key Laboratory of Pollution Processes and Environmental Criteria, Ministry of Education, Nankai University, Tianjin 300350, China

^3^Department of Water and Wastewater Engineering, School of Urban Construction, Wuhan University of Science and Technology, Wuhan 430065, China

*Corresponding author. E-mail: [zhangxiaoyuan@nankai.edu.cn](mailto:zhangxiaoyuan@nankai.edu.cn); [lujinfeng@nankai.edu.cn](mailto:lujinfeng@nankai.edu.cn); [cyliu@nankai.edu.cn](mailto:cyliu@nankai.edu.cn)

The following are included as supporting information for this paper:

number of texts: 6

number of figures: 12

number of tables: 2

number of movies: 1

**Text S1. Preparation of redox-active conductive TA-FeBC carrier**

The redox-active conductive TA-FeBC carrier was prepared by a one-step hydrothermal method as follows: 1 g of biochar (derived from wood waste via pyrolysis at 500°C), 20.0 mg of FeCl_3_·6H_2_O and 75.6 mg tannic acid (TA) were dissolved in 40.0 mL of deionized water. The pH was adjusted to 8.50 using 10 mM NaOH. Carbon felt (CF) was cut into 2 cm × 2 cm × 0.2 cm pieces, immersed in solution and stirred for 12 h. The mixture was placed in a 100 mL hydrothermal reactor and the reaction was sealed at 120 °C for 12 h. After the reaction was completed and cooled to room temperature, it was washed three times with deionized water and dried in a vacuum oven at 60°C to obtain a redox-active conductive TA-FeBC carrier. **Text S2.** **The pretreated procedure of carbon felt**

The pretreated procedure of carbon felt is as follows: a piece of carbon felt of 2 × 2 × 0.2 cm^3^ was immersed rinse the carbon felt removed from the solution to neutral pH with deionized water and ethanol, and drying at 60 °C for 12 h, the CF was obtained for further experiment.

**Text S3. Enrichment and cultivation experiments of biofilms on redox-active conductive TA-FeBC carrier in anaerobic fixed biofilm reactor**

The experiments were carried out in a series of 250 mL of serum bottles with working volumes of 200 mL by semi-continuous feeding mode for 20 operation cycles. The inoculum sludge was obtained from an anaerobic up-flow anaerobic sludge blanket (UASB) reactor that had been steadily operated in the laboratory for one year. After purging with N_2_ to maintain anaerobic conditions, the temperature of each reactor was maintained at 37 ± 0.5°C with a water bath, and the reactors were stirred at 150 rpm. Inoculated anaerobic suspended sludge at a concentration of 5 g VSS/L was used for all groups. The hydraulic retention time (HRT) was set to 12 h. To enable in-depth investigation of the dynamic evolution of biofilm activity and electron transfer behaviors, synthetic municipal wastewater was used as the substrate, containing 500 mg COD/L as carbon source (320 mg/L sodium acetate and 266 mg/L glucose), 25 mg/L NH_4_^+^-N, 5 mg/L PO_4_^3-^-P, 500 mg/L NaHCO_3_, 45 mg/L CaCl_2_·2H_2_O, 10 mg/L MgCl_2_⋅6H_2_O, 10 mg/L FeCl_2_·4H_2_O, and other trace elements (0.12 mg/L ZnSO_4_⋅7H_2_O, 0.03 mg/L CuSO_4_, 0.12 mg/L MnCl_2_·4H_2_O, 0.15 mg/L H_3_BO_3_, and 0.18 mg/L KI) ^[1,2]^.

Total organic carbon (TOC) was measured by sampling at 4, 8 and 12 h until 20 cycles of operation by total organic carbon analyzer (TOC-L, SHIMADZU, Japan). The biogas produced every day was collected into the aluminum foil gas bag and analyzed using a gas chromatograph (GC-8860, Agilent, USA) equipped with a thermal conductivity detector, and the modified Gompertz model was used to fit CH_4_ production data. Methane production from the anaerobic suspended sludge and anaerobic biofilm were measured from control and redox-active conductive TA-FeBC carrier groups, respectively. TS and VS were determined according to the Standard Methods for the Examination of Water and Wastewater (APHA, 2005). The pH values of influent and effluent in all reactors remained stable at 7.5-8.0.

A modified Gompertz model was used to quantitatively analyze methane production.

$P(t)=P_{m}\times\exp\left\{ -\exp\left[ \frac{R_{m}\times e}{P}\times(\lambda-t)+1 \right] \right\}$ (S1)

Where *P(t)* (mL) is the cumulative methane production at time t, *P_m_* (mL) is the maximum methane production potential, *R_m_* (mL/d) is the maximum methane production rate, *λ* (d) is the lag phase time, and e is 2.71828.

**Text S4. I-T test for confirming the hypothesis direct electron transfer (DET)**

DET from the redox-active conductive TA-FeBC carrier to the anaerobic biofilm was evaluated with a two-chamber electrochemical system separated by a proton exchange membrane. The redox-active conductive TA-FeBC carrier was used as the anode and the anaerobic biofilm on glassy carbon electrode (diameter 3 mm) as the cathode, and Ag/AgCl was used as the reference electrode. The volume of each chamber was 30 mL, with a 25-mL liquid volume. I-T curve was monitored under an open-circuit potential condition to depict the increment of cathodic current.

**Text S5. Fluorescence in situ hybridization combined with confocal laser scanning microscopy (FISH-CLSM)**

At the end of the methanogenesis experiment, the anaerobic suspended sludge mixture and redox-active conductive TA-FeBC carrier supported anaerobic biofilm were cleaned three times with PBS solution and then fixed with 4% paraformaldehyde at 4℃ for 12 h. Two probes specific for *Methanobacterium* (Cy3: CGCCATGCCTGACACCTAGCGAGC) and *Methanosaeta* (MX825-FAM: TCGCACCGTGGCCGACACCTAGC) were used. Consecutive scanning was used to detect Cy3 (excitation 550 nm/emission 590 nm) and FAM (excitation 480 nm/emission 530 nm) fluorescence. After fixation, a buffer containing 50 ng/μL specific probe was hybridized with the embedded sections of the samples at 40℃ for overnight. After hybridization, the hybrid was washed with a buffer solution at 37℃. The distribution of specific cells within the anaerobic suspended sludge mixture and anaerobic biofilm were visualized by CLSM (Leica STELLARIS 5, Germany). The image data were processed by Image J software (NIH, Bethesda, MD, USA) to describe fluorescence intensity (FI).

**Text S6. Microbial community and** **metatranscriptomic analysis**

The microbial samples of anaerobic suspended sludge mixture and redox-active conductive TA-FeBC carrier supported anaerobic biofilm were collected at the end of the methanogenesis experiment for the characterization of microbial community structure. The bacterial 16s rRNA gene was amplified using the 338F (5′-ACTCCTACGGGAGGCAGCAG-3′) and 806R (5′- GGACTACHVGGGTWTCTAAT-3′) primer targeting the variable region V3-V4. The universal primer pair 524F10extF (5′-TGYCAGCCGCCGCGGTAA-3′) and Arch958RmodR (5′-YCCGGCGTTGAVTCCAATT-3′) were used for polymerase chain reaction amplification of the archaeal 16S rRNA gene. The representative sequences and ASV abundance tables obtained were then compared with databases to get microbial community structure information. Besides, metatranscriptomic sequencing analysis of anaerobic suspended sludge mixture and anaerobic biofilm was also performed to analyze the functional microbial abundance and gene expression levels. First, total RNA was extracted from the collected biofilm samples using the RNAprep Pure Cell/Bacteria kit. Then, the extracted total RNA was subjected to complex operations such as quality testing, library construction, and Illumina sequencing through Shanghai Majorbio Co., Ltd., and finally, the raw data were obtained. The raw data is then taken through the steps of mixing, assembling, and functional annotation to finally gain the functional gene annotation information and expression levels. The statistical analysis was performed on a free online platform, the Majorbio Cloud Platform.

|  |
| --- |

Movie S1 The wettability of pristine carrier (carbon felt) and redox-active conductive TA-FeBC carrier.


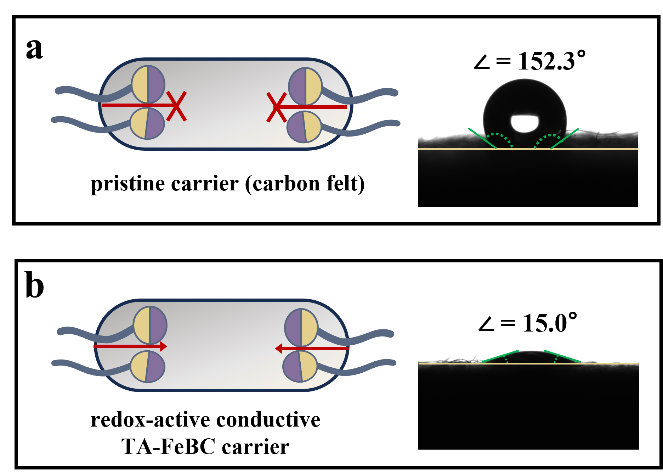


Fig. S1 The photographs of water dispersion on the pristine carrier (carbon felt) (a) and redox-active conductive TA-FeBC carrier (b) and the corresponding water contact angle.


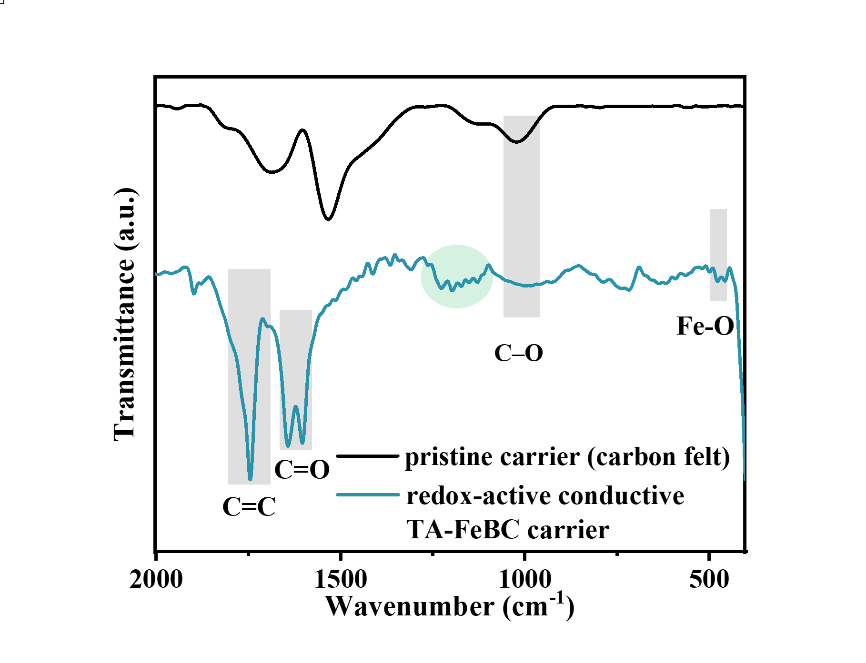


Fig. S2 The FTIR spectra for the pristine carrier (carbon felt) and redox-active conductive TA-FeBC carrier.


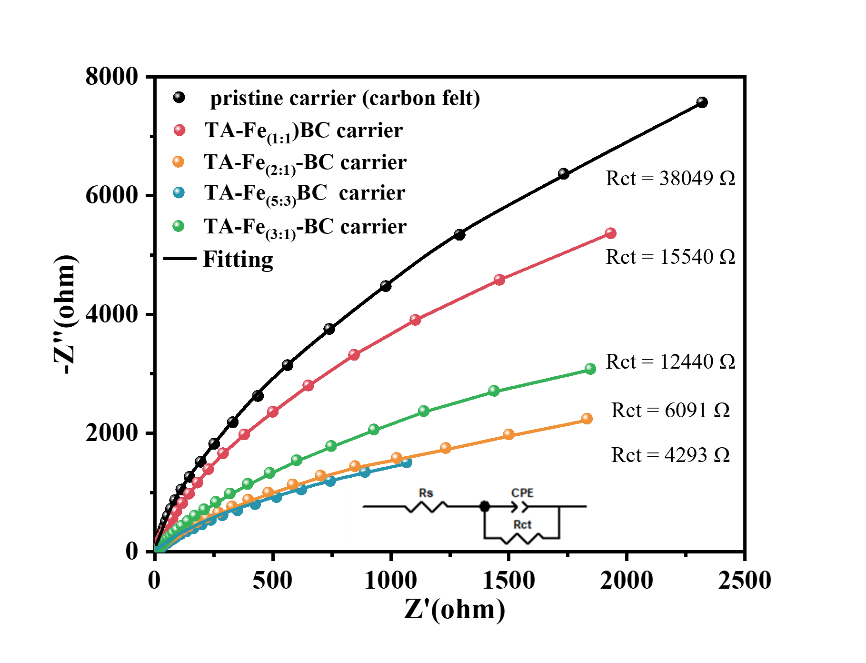


Fig. S3 EIS Nyquist plots with resistance fitted by equivalent circuit for the pristine carrier (carbon felt) and redox-active conductive TA-FeBC carrier with different Fe: TA molar ratio. (The 1:1, 5:3, 2:1, and 3:1 represent the Fe:TA molar ratio of redox-active conductive TA-FeBC carrier.)


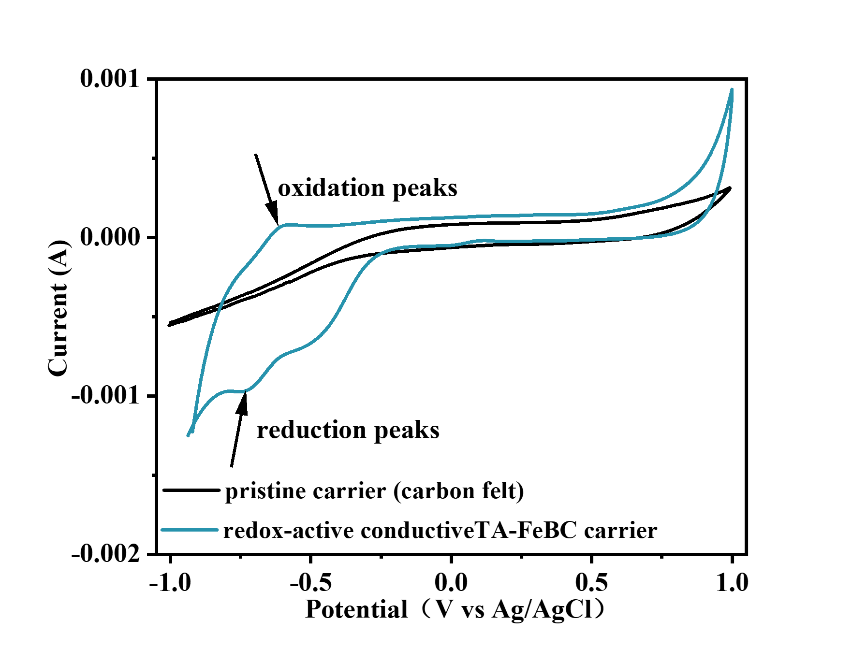


Fig. S4 CV curves for the pristine carrier (carbon felt) and redox-active conductive TA-FeBC carrier.


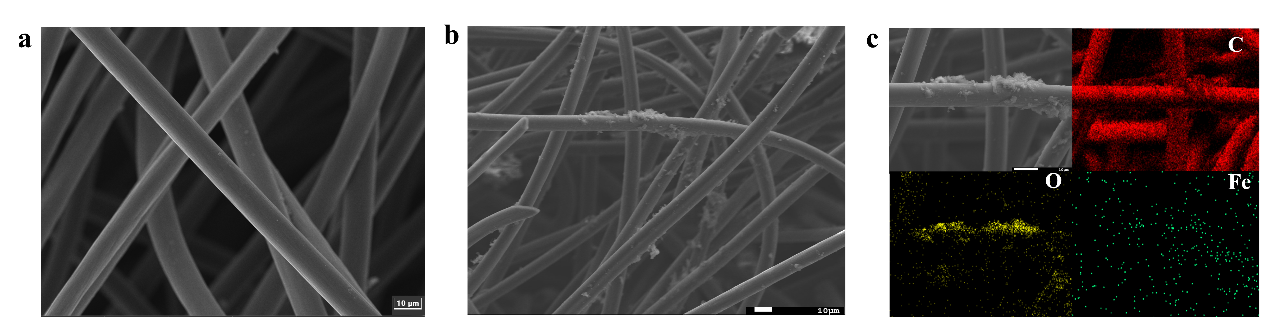


Fig. S5 The SEM images (a) pristine carrier (carbon felt) and (b) redox-active conductive TA-FeBC carrier, (c) EDS mappings of redox-active conductive TA-FeBC carrier.


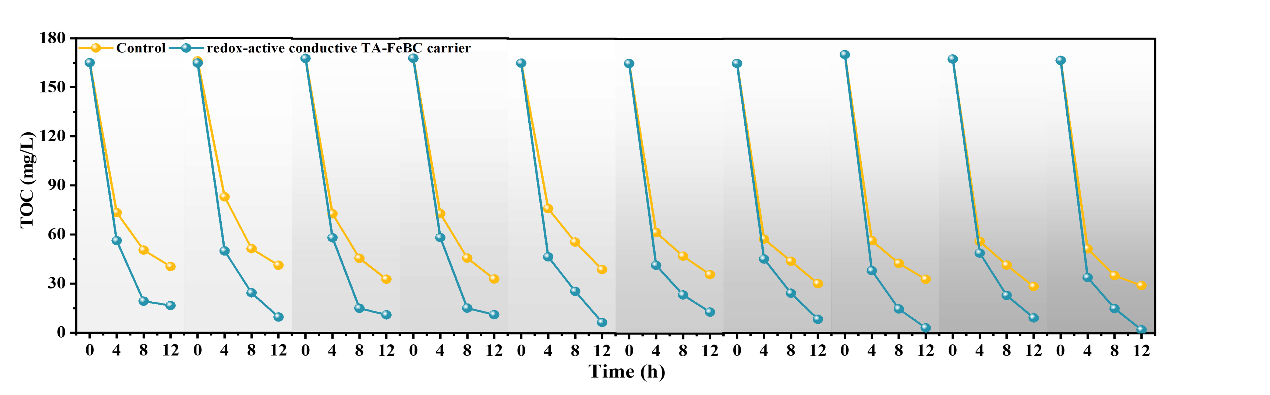


Fig. S6 The variation of TOC from the control (anaerobic suspended sludge) and redox-active conductive TA-FeBC carrier supported anaerobic biofilm reactors.


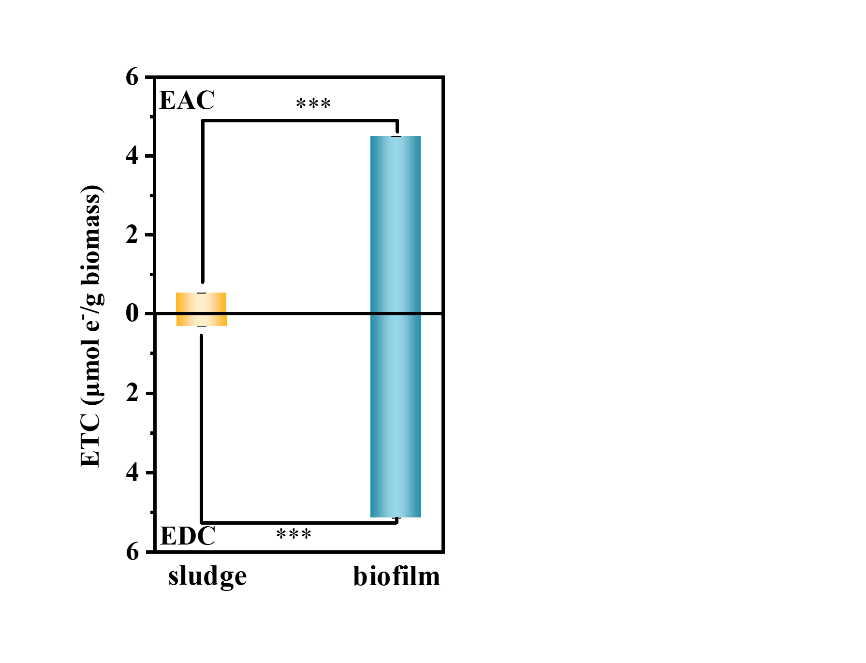


Fig. S7 EAC and EDC of anaerobic suspended sludge and anaerobic biofilm on redox-active conductive TA-FeBC carrier.


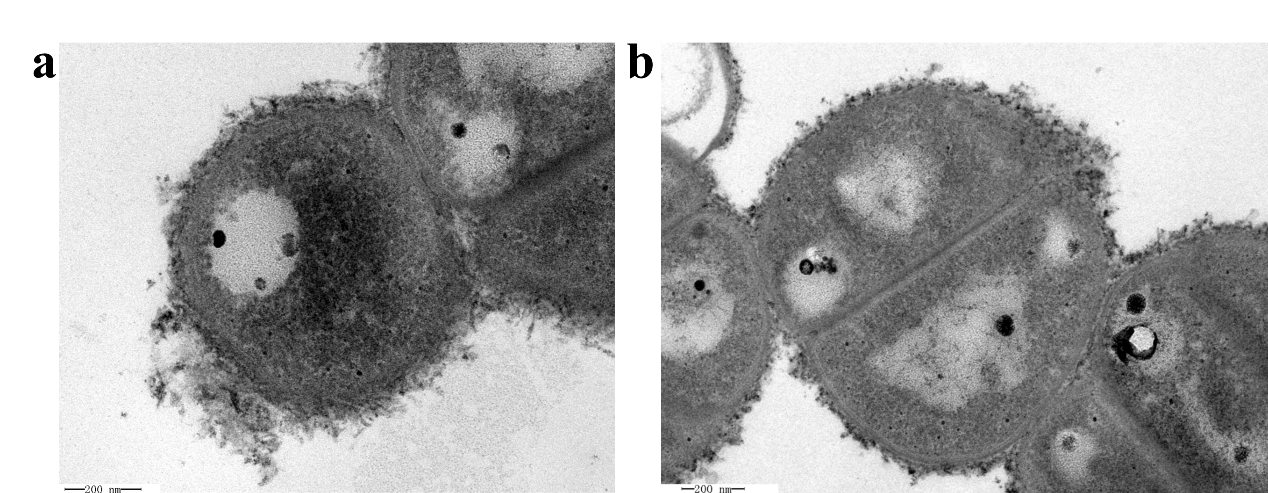


Fig. S8 TEM image (negative DAB staining in the absence of H_2_O_2_) on cell membranes of (a) anaerobic suspended sludge and (b) anaerobic biofilm on redox-active conductive TA-FeBC carrier.


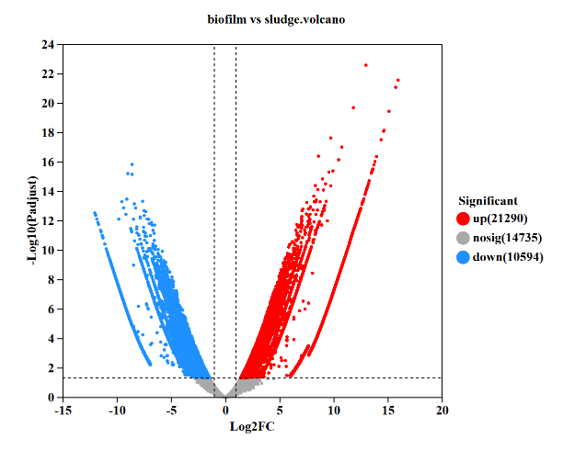


Fig. S9 Volcano map of differential genes (redox-active conductive TA-FeBC carrier: anaerobic biofilm vs. anaerobic suspended sludge).


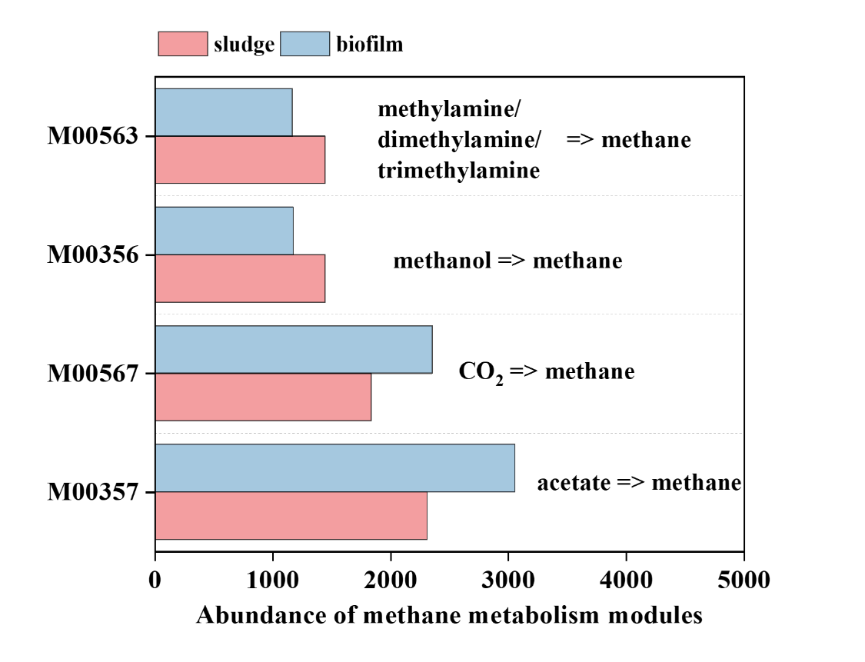


Fig. S10 The transcript abundance of key methane metabolism modules for anaerobic biofilm on redox-active conductive TA-FeBC carrier and anaerobic suspended sludge.


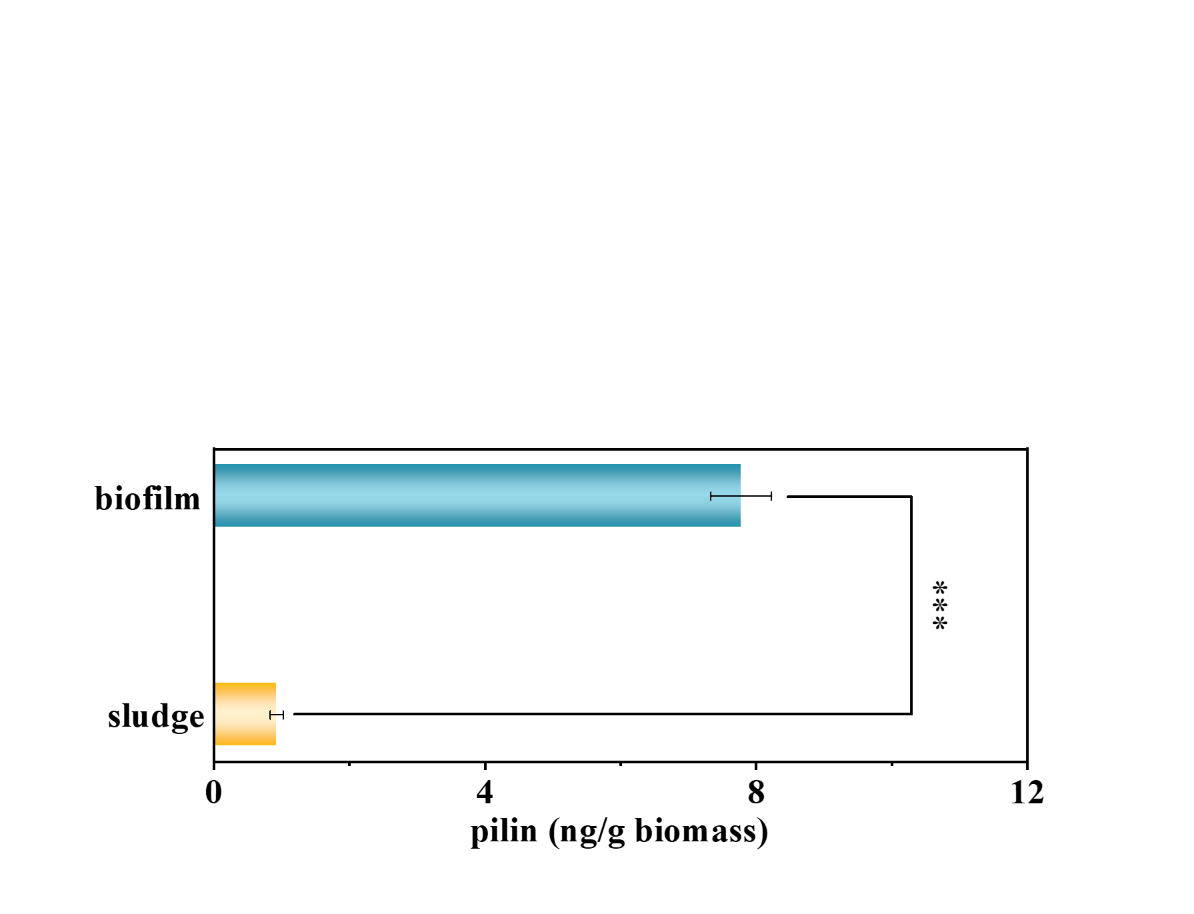


Fig. S11 Pilin concentration of anaerobic biofilm on redox-active conductive TA-FeBC carrier and anaerobic suspended sludge.


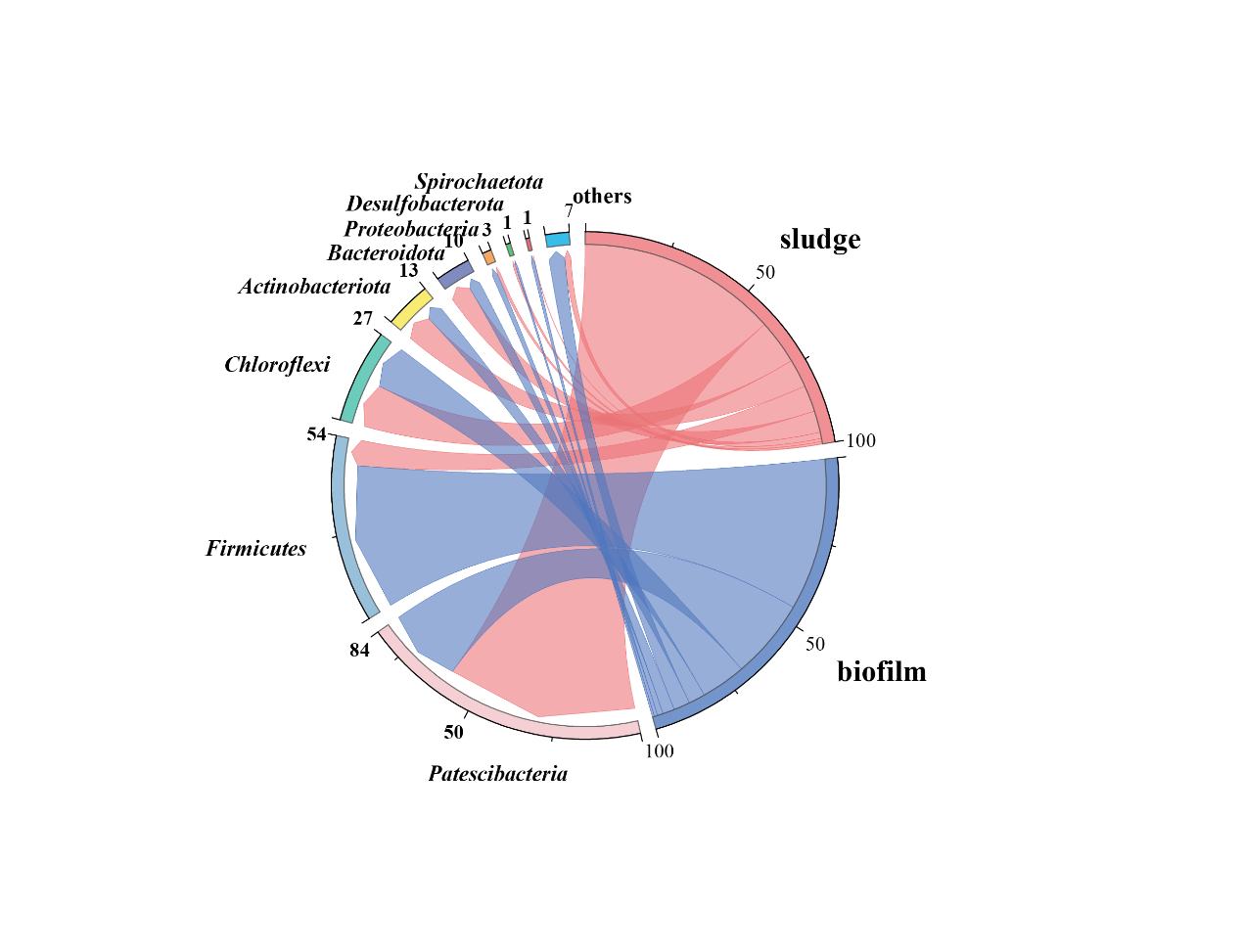


Fig. S12 The community structure analysis of bacteria based on phylum level of anaerobic biofilm on redox-active conductive TA-FeBC carrier and anaerobic suspended sludge (phylum level with relative abundance lower than 1.0% were categorized into others).

The redox-active conductive TA-FeBC carrier exhibited intimate interactions with anaerobic microorganisms in mediating electron transfer. The compositional structure of anaerobic microbiological communities driven by TA-FeBC biofilm was analyzed to gain insights into the underlying mechanisms behind the increased methane yield and electron transfer in anaerobic biofilm reactor. Notably, Firmicutes was selectively enriched in anaerobic biofilm, which provided a temporary electron storage function for methanogens, allowing for the targeted transfer of electrons to the methanogenic process via DIET pathway. The relative abundance of Firmicutes increased from 7.98% of anaerobic suspended sludge to 45.7% of anaerobic biofilm. Thus, redox-active conductive TA-FeBC carrier promoted the enrichment of functional bacteria on the anaerobic biofilm and enhanced the availability of electron storage sites, thereby facilitating DIET at the carrier-biofilm interface.

Table S1. Kinetic parameters of the methane production by fitting the modified Gompertz equation.

|  | ***P*_m_ (mL)** | ***R*_m_ (mL/h)** | ***λ* (h)** | **R^2^** |
| --- | --- | --- | --- | --- |
| **Control (anaerobic suspended sludge)** | 180±13.9 | 0.651±0.0205 | 26.1±3.49 | 0.996 |
| **Anaerobic biofilm on redox-active conductive TA-FeBC carrier** | 387±16.1 | 1.69±0.0517 | 18.6±4.31 | 0.998 |

Table S2. Charge Calculation Based on DPV.

|  | **Peak** | **Potential/V** | **Current/mA** | **Q_DPV_/C** | ***Q*_formal_/C** |
| --- | --- | --- | --- | --- | --- |
| **Anaerobic biofilm on redox-active conductive TA-FeBC carrier** | CytC | -0.1 | 1.16 | 1.96×10^-4^ | 8.87×10^-2^ |
|  | Flavins/CytC combination | -0.300 | 2.17 | 3.08×10^-4^ | 1.39×10^-1^ |

**References**

1 Zhang, X., Gu, J. & Liu, Y. Necessity of direct energy and ammonium recovery for carbon neutral municipal wastewater reclamation in an innovative anaerobic MBR-biochar adsorption-reverse osmosis process. *Water Res.* **211**, 118058, (2022).

2 Wang, Z. *et al.* Enhanced treatment of low-temperature and low carbon/nitrogen ratio wastewater by corncob-based fixed bed bioreactor coupled sequencing batch reactor. *Bioresource Technol.* **351**, 126975, (2022).
